# An A549 Cell-Based Approach Using Repeated Fluorescence Readouts for Assessing Reactive Oxygen Species Activity of Atmospheric Particulate Matter

**DOI:** 10.3390/toxics14090789

**Published:** 2026-09-07

**Authors:** Ioanna Tzagkaroulaki, Evangelia Diapouli, Vasiliki Vasilatou, Stefanos Papagiannis, Efthimios Tagaris

**Affiliations:** 1 National Centre for Scientific Research ‘Demokritos’, Institute of Nuclear & Radiological Sciences & Technology, Energy & Safety, Agia Paraskevi, 15341 Athens, Greece; vassiliki@ipta.demokritos.gr (V.V.); s.papagiannis@uoi.gr (S.P.); 2 Department of Chemical Engineering, University of Western Macedonia, Active Urban Planning, 50150 Kozani, Greece; etagaris@uowm.gr; 3 Department of Materials Science & Engineering, University of Ioannina, 45110 Ioannina, Greece

**Keywords:** reactive oxygen species, oxidative stress, particulate matter, positive control, biological systems, human cells

## Abstract

Exposure to atmospheric particulate matter (PM) is a major public-health concern, in part because PM can perturb cellular redox homeostasis. This study evaluates an in vitro A549/DCFH-DA approach using repeated fluorescence readouts to assess PM2.5-induced oxidative activity. Untreated and assay-specific controls were combined with zymosan and NIST Standard Reference Material^®^ 2584 suspended in PBS, and fluorescence was monitored at multiple readout times over a 15 min–6 h window. Method performance was characterized using the coefficient of variation (CV) and signal-to-noise ratio (SNR). A dedicated three-concentration SRM 2584 series (0.02, 0.05 and 0.10 mg mL^−1^) further showed readout-dependent concentration behaviour: at 60 min the untreated-control-corrected mean response increased across the tested concentrations and followed an approximate descriptive linear trend (R^2^ = 0.90), whereas earlier readouts were non-monotonic. Substrate-related effects were examined using paired PTFE and quartz filters. Among the eight matched PTFE–quartz pairs included in the regression analysis, zero-intercept fits showed slopes close to unity for both mass- and air-volume-normalized responses (0.90 and 0.99, respectively; R^2^ ≈ 0.99), demonstrating strong proportional agreement within this comparison set; the limited number of pairs does not support universal substrate interchangeability. Application to chemically characterized field PM2.5 samples from an urban-background site and a high-altitude site showed that DCFH-DA fluorescence did not track PM mass alone and is interpreted in terms of exploratory associations with particle composition, rather than causal effects of individual constituents. Taken together, these findings support the use of the method-performance-characterized workflow for assessing oxidative responses to field-collected PM2.5 across multiple readout times and for investigating their associations with particle chemical characteristics.

## 1. Introduction

Air pollution remains a major environmental determinant of public health, with particulate matter (PM) among its most harmful components. The World Health Organization reports widespread exposure above recommended air-quality guidelines and millions of premature deaths associated with polluted air [1]. Epidemiological and toxicological evidence further show that PM health effects are not explained by particle mass alone, but also by particle size, surface area, chemical composition and source-related properties [2,3,4,5].

Atmospheric aerosols comprise primary particles emitted directly from natural and anthropogenic sources and secondary particles formed through atmospheric oxidation, nucleation, condensation and multiphase reactions. PM10 and PM2.5 denote particles with aerodynamic diameters below 10 and 2.5 μm, respectively [6]. Fine particles are of particular concern because they can reach the alveolar region, while their atmospheric behaviour and biological impact depend on interconnected physicochemical properties, including size, morphology, hygroscopicity, surface area and chemical composition [7,8,9,10,11].

The mechanisms by which PM causes adverse health effects are still not fully resolved, but oxidative stress is widely considered a central pathway. Inhaled particles may deposit in the airways and alveoli, interact with epithelial cells and resident immune cells, and trigger inflammatory and redox-active processes [12]. PM may also carry transition metals, inorganic ions, organic compounds, quinones, mineral dust, elemental and organic carbon, and biologically derived material, all of which can contribute differently to particle reactivity. When the production of reactive oxygen species (ROS) exceeds the capacity of the cellular antioxidant defence system to neutralize reactive intermediates or repair the resulting damage, oxidative stress can occur. This process is directly connected with pulmonary inflammation, cytotoxicity, DNA oxidation, mutagenicity and pro-inflammatory signalling [13,14]. For this reason, the oxidative potential and ROS-generating activity of PM have become increasingly important endpoints for assessing the toxicological relevance of atmospheric particles beyond gravimetric mass concentration alone.

Several acellular assays have been developed to assess the oxidative potential of PM in a cell-free environment. Common approaches include electron spin or electron paramagnetic resonance, the dithiothreitol assay, the ascorbic acid assay and the glutathione assay [15]. These methods are useful because they are relatively simple, reproducible and suitable for the analysis of large sample sets, including source-apportionment and exposure-assessment studies [16]. They can also provide information on the contribution of redox-active metals and oxidized organic compounds to the depletion of chemical reductants or antioxidants. Several studies have suggested that acellular oxidative-potential metrics may be more strongly associated with selected cardiorespiratory outcomes than PM mass alone [17]. At the same time, different acellular assays respond differently to particle composition: some are particularly sensitive to metals such as copper and iron, whereas others respond more strongly to organic aerosol components, including photochemically aged or biomass-burning organic aerosols [18,19].

Despite their usefulness, acellular methods cannot fully reproduce the biological complexity of a living cellular system. They do not capture cell-mediated ROS generation, mitochondrial responses, activation of membrane receptors, inflammatory signalling, antioxidant defence mechanisms, repair processes or adaptive responses. This limitation is particularly important because PM-induced ROS in cells can arise not only from the intrinsic chemical reactivity of the particles, but also from cellular processes triggered after exposure [20]. Therefore, acellular assays are best regarded as complementary tools that can screen PM redox activity and identify potentially reactive particle components, while cell-based systems provide a more physiologically relevant assessment of the oxidative response induced by PM in biological models.

Cellular assays use viable cells to evaluate the biological response to particles or particle extracts. In the context of PM toxicology, such assays can integrate both chemically generated ROS and ROS produced by cells after particle stimulation [21]. Fluorescence-based probes, including luminol, 2′,7′-dichlorodihydrofluorescein diacetate (DCFH-DA) and dihydrorhodamine 123, have been widely applied for assessing oxidative responses in pulmonary cell systems [22]. The choice of cellular model is critical because different cell types may respond to PM through different mechanisms. Respiratory-tract cells are commonly used because inhalation is a primary route of exposure. Among the most frequently used models are A549 human alveolar epithelial cells and NR8383 rat alveolar macrophages [23,24,25]. Macrophage-like models are highly responsive to particle-induced inflammatory and oxidative stimuli, whereas epithelial models are relevant for studying the interaction of PM with the lung barrier and for evaluating oxidative stress, cytotoxicity and epithelial-cell responses [26,27]. A549 cells are widely used in in-vitro toxicology because they are robust, reproducible, easy to culture, and representative of key features of human lung epithelial response [28,29].

Control strategies are essential for reliable interpretation of in vitro ROS assays. Defined chemical oxidants (e.g., tert-butyl hydroperoxide), biological stimuli such as zymosan, and PM-relevant reference materials serve complementary purposes: a soluble oxidant primarily verifies assay responsiveness, whereas a particulate reference material challenges more of the extraction/exposure/cell/probe workflow. Zymosan has been widely used as a positive biological stimulus in oxidative-burst assays [30]. Reference dusts and ambient PM, in contrast, comprise complex mixtures of metals, organics and other redox-active constituents that more closely resemble environmental particle exposures [31]. Standard dust materials can therefore provide useful PM-relevant performance references [32,33,34]. In the present study, SRM 2584 was selected as the primary particulate reference control, while zymosan was retained as a comparator. A classical soluble chemical oxidant was not included in the experimental series; this scope choice is discussed in Section 3.6.

Assay-specific controls are equally important because fluorescence-based measurements may be affected by background fluorescence, particle autofluorescence, optical interference, probe oxidation in the absence of cells, fluorescence quenching and medium-related effects. DCFH-DA is cell-permeable and is widely used as a broad redox-sensitive probe [22,35,36]. However, DCFH/DCF chemistry is not selective for a single ROS, can be influenced by cellular and chemical context, and should not be interpreted as a species-specific quantitative ROS measurement [37,38]. We therefore interpret the readout as an integrated cellular oxidative-response signal and include untreated, blank-filter, no-cell and no-probe controls to identify non-specific assay contributions [33].

**Scheme 1:**
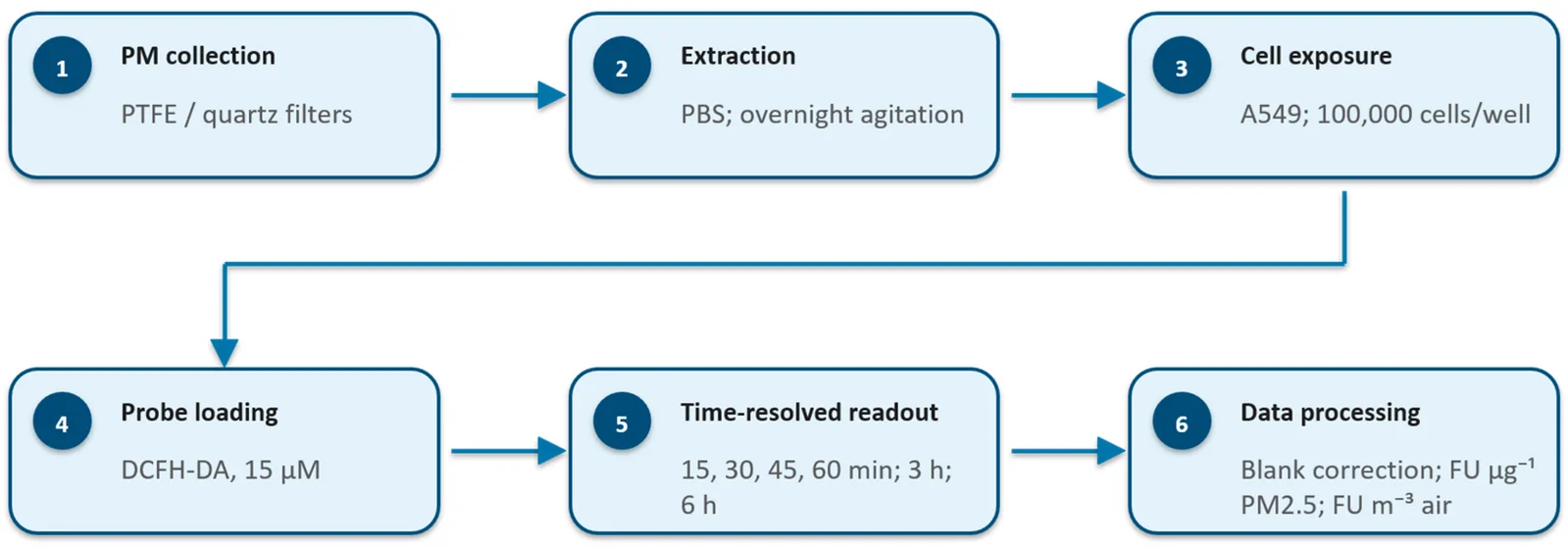
Experimental workflow for the A549 cell-based PM oxidative-response assay with repeated fluorescence readouts.

The present study evaluates an A549 cell-based approach using repeated fluorescence readouts to assess PM-induced oxidative activity with DCFH-DA. The contribution does not lie in the use of A549 cells or DCFH-DA alone, both of which are established, but in the integration of several methodological elements within one filter-based workflow: assay-specific controls; comparison of zymosan with a PM-relevant NIST reference dust; fluorescence characterization across an extended 15 min–6 h kinetic series; explicit evaluation of PTFE and quartz filter substrates after blank correction; and reporting on both particle-mass- and sampled-air-volume-normalized bases. The characterized workflow (see also Scheme 1) is then applied to chemically characterized ambient PM samples. Because oxidative-potential assays remain method-dependent and are not yet harmonized to a single universal reference method [39], this work is presented as method-performance characterization and field application, rather than as validation against a gold-standard assay. A further objective was to determine whether the characterized workflow could discriminate oxidative-response profiles among field-collected PM2.5 samples across multiple readout times and thereby provide a basis for investigating relationships between cellular oxidative activity and particle composition.

## 2. Materials and Methods

### 2.1. Stations and Sample Collection

**Figure 1:**
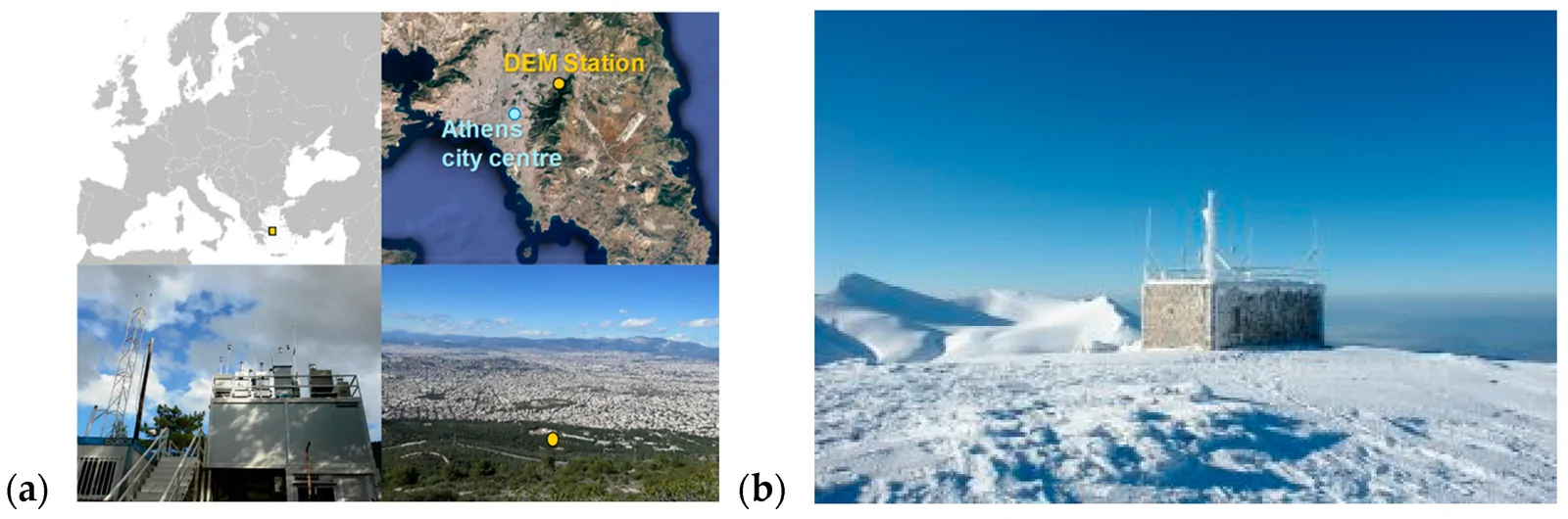
(**a**) The DEM urban background station in Ag. Paraskevi, Athens, Greece (37.995 N 23.816 E, at 270 m above sea level); the yellow marker indicates the location of the DEM station. (**b**) The Helmos Hellenic Atmospheric Aerosols and Climate Change Station (HAC)^2^ at an altitude of 2324 m.

The PM filters analysed in this study were obtained from two established atmospheric monitoring sites in Greece (Figure 1): the Demokritos Atmospheric Aerosol Measurement urban-background station in Agia Paraskevi, Athens, Greece (DEM), and the Helmos Hellenic Atmospheric Aerosols and Climate Change Station (HAC)^2^, a high-altitude site in Greece [40]. These stations represent contrasting atmospheric environments relevant to PM characterization. Detailed descriptions of the sites, their operation and their atmospheric context are provided elsewhere [41,42].

Three field-filter series were included in the study. The first series consisted of PTFE PM2.5 filters collected at DEM during 2013. The second series consisted of PTFE PM2.5 filters collected at the same station from September 2019 to September 2020, covering seasonal variability. The third series consisted of quartz-fibre filters collected at (HAC)^2^ during the sampling periods included in the field-sample analysis. DEM samples represented 24 h collections. The (HAC)^2^ samples span the collection intervals listed in Table 4; because collection duration and sampling configuration differed from DEM, cross-site comparisons are descriptive. For the 2019–2020 DEM series, 24 h PM2.5 samples were collected every three days using a low-volume reference sampler (Sequential 47/50-CD, Sven Leckel GmbH, Berlin, Germany) operated at 2.3 m^3^ h^−1^. Field or laboratory blanks represented approximately 6% of the total number of filters and were handled identically to the corresponding samples. At (HAC)^2^, samples were collected on 150 mm high-purity, binder-free Tissuquartz quartz-fibre filters (Pall Corporation, Port Washington, NY, USA), using a standard high-volume sampler (Model DHA-80, Digitel Enviro-Sense GmbH, Weingarten, Germany) operated at a nominal flow rate of 500 L min^−1^. Corresponding quartz-fibre blank filters were handled and processed identically to the field samples.

### 2.2. Filters

Two filter substrates commonly used in PM characterization were considered: PTFE (Teflon) filters, which are suitable for gravimetric PM mass determination and elemental analysis, and Quartz fibre filters, which are required for carbonaceous aerosol analysis (OC/EC). The PTFE filters used at the DEM station were 47 mm PTFE (polypropylene-backed) filters (Whatman/Cytiva, Marlborough, MA, USA). The Quartz fibre filters used at (HAC)^2^ station were 150 mm high-purity, binder-free Tissuquartz 2500 QAT-UP filters (Pall Corporation, Port Washington, NY, USA), with a nominal thickness of 432 μm. According to the manufacturer’s specifications, the filters have an aerosol-retention efficiency of 99.9% for 0.3 μm dioctyl phthalate particles, determined according to ASTM D 2986-95A [43] at a test flow rate of 32 L min^−1^ per 100 cm^2^. Gravimetric mass determination followed EN12341 and standard conditioning/weighing procedures [44]. Filters were conditioned for 48 h before and after sampling under controlled temperature and relative humidity conditions, and were weighed by the use of a microbalance (d = 0.01 mg).

### 2.3. Chemical Analyses

Each sample and blank filter was sectioned into two parts: one half was used for ROS bioassay extraction and the other half for chemical analysis. PM mass concentration was calculated from filter load and sampled air volume. Chemical characterization was performed to support the interpretation of ROS responses in relation to PM composition. Major and trace elements were quantified by energy-dispersive X-ray fluorescence (ED-XRF) following previously published procedures. Both Teflon and Quartz fibre filters were analysed. Additional details on analytical and quality assurance/quality control procedures are available in the cited protocols [45,46,47,48].

Organic carbon (OC) and elemental carbon (EC) were determined by thermo-optical transmittance using the EUSAAR2 protocol, with charring correction based on transmittance monitoring. At (HAC)^2^ station, the collected Quartz fibre filters were analysed by the use of a lab analyser (Lab OC-EC Aerosol Analyzer, Sunset Laboratory, Inc., Tigard, OR, USA). Detailed analytical procedures are described in the cited methodology [47]. At DEM station, a field analyser was deployed (OC-EC field analyser, Model-4, Sunset Laboratory, Inc., Tigard, OR, USA) for the near-real-time monitoring of elemental (EC) and organic carbon (OC) concentrations in PM2.5, on a 3 h basis [49]. The 3 h data were averaged over the 24 h period corresponding to the sampling time of the PTFE (Teflon) filters collected at DEM station.

The elemental and carbonaceous-composition data were subsequently used to examine variations in ROS responses across the repeated readout times in relation to PM mass, major and trace elements, and the OC and EC fractions.

### 2.4. Cell Culture

Experiments were performed using the human lung adenocarcinoma epithelial A549 cell line (Cellosaurus CVCL_0023; RRID:CVCL_0023), kindly provided by Dr Dimitris Kletsas (Institute of Biosciences and Applications, National Centre for Scientific Research “Demokritos”, Athens, Greece). The cells were used as an established A549 model and maintained under the culture conditions described below, in accordance with general cell-line reporting and good-practice guidance [50]. Cells were cultured in Dulbecco’s Modified Eagle Medium (Biowest, Nuaillé, France), containing L-glutamine and supplemented with 10% heat-inactivated foetal bovine serum (Gibco, Grand Island, NY, USA) and penicillin/streptomycin (Biowest) at final concentrations of 100 U mL^−1^ and 100 μg mL^−1^, respectively. Cultures were maintained at 37 °C in a humidified 5% CO_2_ incubator and subcultured approximately every three days using 0.25% Trypsin-EDTA (Biowest).

### 2.5. Filter Preparation for ROS Bioassay

For ROS bioassay extraction, the half-filter portions were placed in sterile microcentrifuge tubes and PBS was added (968 µL for PTFE filters from DEM and 686 μL for quartz filters from (HAC)^2^), following the extraction-volume rationale described by Landreman et al. [33]. The tubes were maintained overnight in the dark at room temperature under continuous agitation. The next day, samples were centrifuged at 1100 rpm for 1 min and the supernatant was transferred to a new sterile tube; this clarification step was repeated three times to minimize transfer of filter fibres. Field and laboratory blank filters were processed identically. PBS was the routine extraction/exposure vehicle for the field-filter extracts in the present A549 workflow. SGM was used only in the dedicated zymosan-comparator experiments, because it is the simple exposure medium used in the foundational macrophage protocol and permits a direct methodological comparison [33].

### 2.6. ROS Bioassay

A549 cells were seeded in black, clear-bottom 96-well plates (Thermo Fisher Scientific, Waltham, MA, USA) at 100,000 cells per well and incubated for 24 h. The culture medium was then removed and replaced with 100 μL of filter extract or the corresponding control solution. Each condition was measured in four technical replicate wells. Cells were exposed for 2 h 15 min before DCFH-DA (Sigma-Aldrich, St. Louis, MO, USA) was added to a final concentration of 15 μM; the first fluorescence measurement was acquired 15 min later, corresponding to 2.5 h of total particle exposure. Fluorescence was subsequently recorded at 15, 30, 45 and 60 min and, for the extended kinetic series, at 3 and 6 h after probe addition using a Tecan Spark microplate reader (Tecan Austria GmbH, Grödig, Austria) in fluorescence top-reading mode (excitation 450 nm; emission 530 nm; 20 nm excitation and emission bandwidths; 30 flashes; 40 μs integration time; SPARKCTL/MAGELLAN V1.2, Tecan Austria GmbH, Grödig, Austria). Subsequent readouts were collected from the same plate and wells without replacing the exposure medium. Detector gain was set using the instrument’s “Optimal” function at each readout, and therefore varied between readout times. The mean and SD of the four technical wells were calculated for each sample and control at each readout time. The following cellular and assay-specific controls were included in quadruplicate:

(a) wells without cells but with medium and DCFH-DA solution;

(b) wells with medium, without cells, and without DCFH-DA solution;

(c) cells with PBS and DCFH-DA solution;

(d) cells with PBS but without DCFH-DA solution;

(e) cells with NIST SRM 2584 (0.10 mg mL^−1^, suspended in PBS) as the PM-relevant particulate reference control and DCFH-DA solution;

(f) cells with NIST SRM 2584 (0.10 mg mL^−1^, suspended in PBS) but without DCFH-DA solution;

(g) cells with zymosan (0.125 mg mL^−1^) in PBS and DCFH-DA solution; and

(h) cells with zymosan (0.125 mg mL^−1^) in 1× SGM and DCFH-DA solution.

At each fluorescence readout time, the mean fluorescence intensity of the four replicate wells was calculated for each sample, filter blank, and assay control. Sample fluorescence values were corrected using the response of the corresponding field or laboratory blank filter. The resulting blank-corrected ROS activity was expressed on both a particle-mass-normalized and an air-volume-normalized basis. For the DEM samples, the results were expressed as fluorescence units per microgram of collected PM2.5 (FU µg^−1^ PM2.5) and fluorescence units per cubic metre of sampled air (FU m^−3^ air). For the (HAC)^2^ samples, the corresponding mass-normalized results were expressed as FU µg^−1^ PM2.5, together with FU m^−3^ air. These calculations were performed separately for each fluorescence readout time.

### 2.7. Statistical Analysis

Across the study, *n* = 10 independent cell-culture experiments were conducted. Within each independent experiment, each sample and assay-control condition was measured in four technical replicate wells. Results for each condition and fluorescence readout time were summarized as the arithmetic mean and standard deviation (SD). Within-plate technical repeatability was expressed as the coefficient of variation, CV (%) = 100 × SD/mean. Assay sensitivity for each positive/reference control was summarized by the signal-to-noise ratio (SNR), calculated at each readout time as the untreated-control-corrected mean positive/reference-control fluorescence divided by the SD of the corresponding untreated-control wells. CV and SNR values obtained at different readout times were summarized descriptively; different time points were repeated measurements of the same wells, and were not treated as independent biological replicates. For the PTFE–quartz comparison, eight date-matched pairs were analysed. Proportional agreement was evaluated by linear regression constrained through the origin, using the paired maximum blank-corrected responses. Because the field-filter series comprised chemically distinct environmental samples and the number of samples available for composition–ROS comparisons was limited, these relationships were interpreted as exploratory associations, and were not used to infer causality for individual PM constituents. The dedicated SRM 2584 concentration-series experiment comprised one independent cell-culture experiment with *n* = 4 technical wells per concentration. Concentration effects were examined descriptively at 15, 30, 45 and 60 min using untreated-control-corrected mean fluorescence. Because only three non-zero concentrations were tested in this dedicated experiment, linear regression was used only as a descriptive measure of the 60 min trend; no inferential significance, formal analytical linear range or upper detection limit was assigned.

### 2.8. SRM 2584 Concentration-Series Experiment

To characterize concentration–response behaviour of the particulate reference control, A549 cells were exposed to NIST SRM 2584 suspended in PBS at 0.02, 0.05 and 0.10 mg mL^−1^ (20, 50 and 100 μg mL^−1^). The untreated PBS + DCFH-DA cell control was measured in parallel. All other cell-culture, DCFH-DA, and fluorescence-readout conditions were identical to those described above. The concentration series was conducted as one dedicated independent cell-culture experiment, with *n* = 4 technical replicate wells for each concentration and control. Responses were evaluated at 15, 30, 45 and 60 min as the mean fluorescence of each SRM 2584 condition after subtraction of the corresponding untreated-control mean. The concentration ordering was examined at each readout. Because the three-dose series showed a monotonic ordering only at 60 min, a descriptive least-squares linear regression across 0.02–0.10 mg mL^−1^ was used to summarize the 60 min trend. The experiment was not used to define a formal instrumental detection limit or a universally valid analytical linear range.

## 3. Results and Discussion

### 3.1. Assay Controls and Method-Performance Characterization

**Figure 2:**
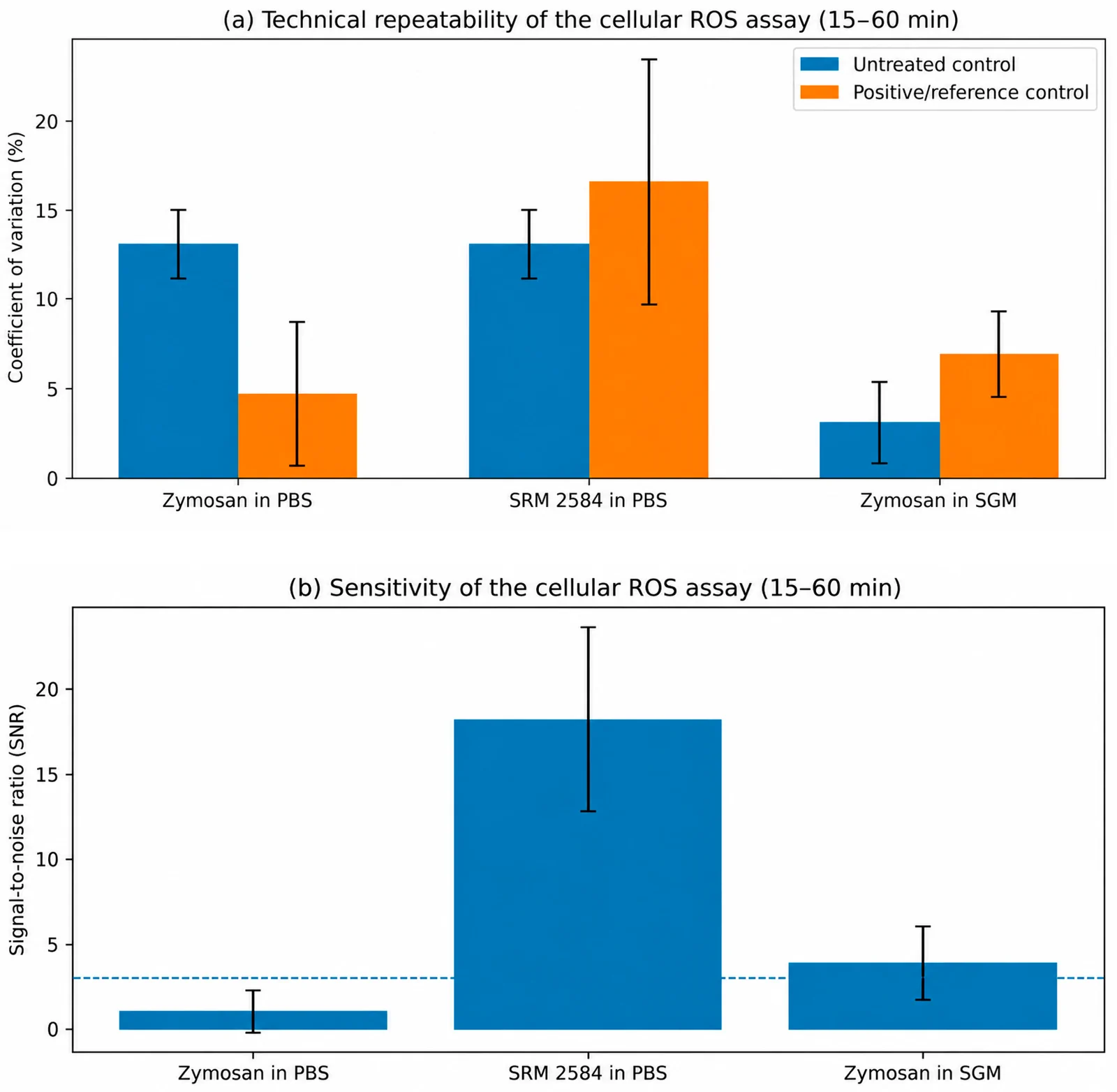
Method-performance summary for the 15–60 min window. (**a**) Mean coefficient of variation (CV) for untreated (UT) and positive/reference-control (POS) conditions; error bars show SD across the four repeated readout times. (**b**) Mean signal-to-noise ratio (SNR) for each stimulus; error bars show SD across the four repeated readout times and the dashed horizontal line indicates SNR = 3 as a descriptive reference. These summaries are descriptive across time points, and do not represent independent biological replicates.

The cellular method-performance analysis used three stimulus conditions: (i) zymosan in PBS, (ii) zymosan in SGM and (iii) NIST SRM 2584 in PBS. No-cell and no-probe wells were assay-specific artefact controls, and were not treated as a separate acellular oxidative-potential method. Within-plate technical repeatability and assay sensitivity are summarized in Figure 2 and Table 1.

Within-plate technical repeatability was quantified using the coefficient of variation (CV) for untreated and positive/reference-control wells, while assay sensitivity was quantified using the signal-to-noise ratio (SNR). For each readout, SNR was calculated as the untreated-control-corrected mean positive/reference-control fluorescence divided by the standard deviation (SD) of the corresponding untreated-control wells. Because the same experimental plate was read repeatedly over time, the readout times were treated as repeated conditions, rather than independent biological replicates.

$$\mathrm{SNR}=\frac{\mathrm{untreated}\text{-}\mathrm{control}\text{-}\mathrm{corrected}\;\mathrm{mean}\;\mathrm{fluorescence}\;\mathrm{of}\;\mathrm{positive}/\mathrm{reference}\;\mathrm{control}}{\mathrm{SD}\;\mathrm{of}\;\mathrm{corresponding}\;\mathrm{untreated}\text{-}\mathrm{control}\;\mathrm{fluorescence}}$$

In this study, CV (%) was used as a descriptive measure of within-plate technical repeatability for untreated and positive/reference-control wells over the initial 15–60 min window. For PBS-based conditions, the untreated-control CV ranged from 11.4 to 15.6% (mean 13.0%); zymosan in PBS showed 1.7–10.4% (mean 4.7%); and SRM 2584 in PBS showed 10.5–25.9% (mean 16.6%). For SGM-based conditions, the untreated-control CV ranged from 0.68 to 6.11% (mean 3.1%) and zymosan in SGM from 3.93 to 9.79% (mean 6.9%). Calculation from the original plate-reader output gave SNR values of −0.71, 1.47, 1.36 and 2.10 for zymosan in PBS; 10.95, 21.33, 17.34 and 23.20 for SRM 2584 in PBS; and 4.91, 6.40, 2.56 and 1.66 for zymosan in SGM at 15, 30, 45 and 60 min, respectively. These data show strong sensitivity for the PM reference material, a moderate and time-dependent response for zymosan in SGM, and a weak early-window response for zymosan in PBS.

The 15–60 min performance analysis showed that assay sensitivity depended strongly on the control stimulus and exposure medium. SRM 2584 in PBS provided the clearest PM-relevant response, with a mean SNR of 18.2 (range 10.95–23.20). Zymosan in SGM produced a moderate and time-dependent response, with a mean SNR of 3.88 (range 1.66–6.40), whereas zymosan in PBS remained comparatively weak within this early window, with a mean SNR of 1.05 (range −0.71–2.10). Accordingly, the data support strong sensitivity to the particulate reference material but do not justify describing every positive-control condition as having SNR > 3. The CV results characterize within-plate technical repeatability of the technical-well readouts, with particularly stable untreated SGM backgrounds.

Within the initial 15–60 min performance window, 30 min provided a strong and practical early readout for SRM 2584 (SNR ≈ 21.3) and zymosan in SGM (SNR ≈ 6.4). However, it was not a universal optimum: zymosan in PBS remained weak, and the extended time course showed later increases for several conditions. We therefore use 30 min only as a standardized early quality-control checkpoint, not as the universal biological maximum for environmental PM samples.

SRM 2584 was retained as the primary PM-relevant reference because, unlike a soluble chemical oxidant, it challenges the particulate-exposure workflow under conditions closer to those used for the environmental extracts [32,51]. The initial 15–60 min series demonstrated that the reference dust was readily distinguishable from the untreated control throughout the early window. This role should not be interpreted as replacing every possible assay-control category; in particular, a classical soluble oxidant such as t-BHP was not included in this experimental series.

**Figure 3:**
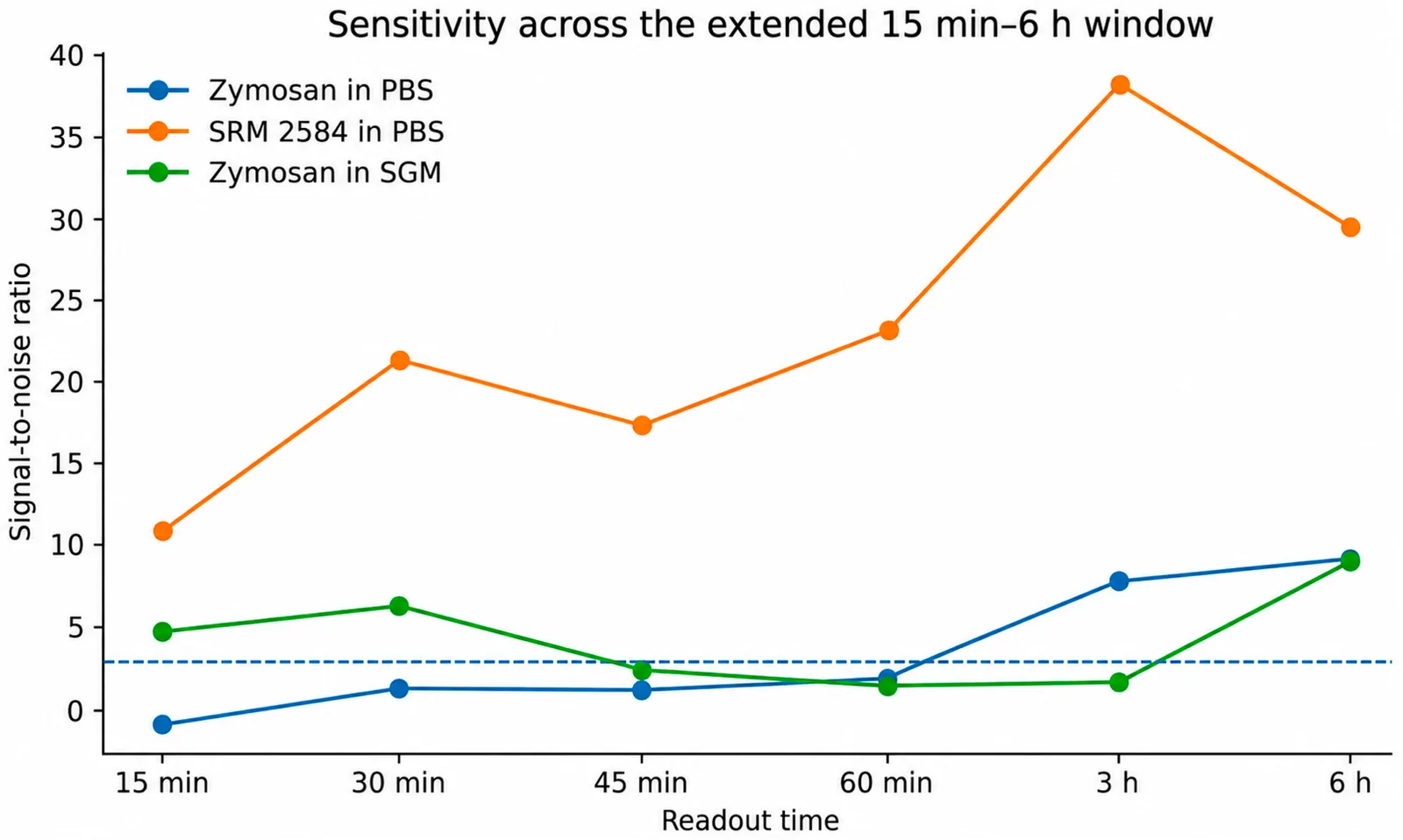
Signal-to-noise ratio (SNR) across the extended 15 min–6 h window. SNR is shown for zymosan in PBS, SRM 2584 in PBS and zymosan in SGM at each readout time. Each value was calculated from *n* = 4 technical wells and the SD of the corresponding untreated-control wells after untreated-control subtraction and the applicable dilution correction. The same wells were read repeatedly over time; therefore, readout times are repeated conditions, rather than independent biological replicates. The dashed horizontal line indicates SNR = 3.

The extended 15 min–6 h dataset is important because it demonstrates stimulus-dependent kinetics, rather than a single common optimum (Figure 3). SRM 2584 reached SNR ≈ 38.3 at 3 h before declining to ≈29.5 at 6 h; zymosan in PBS increased to ≈7.8 and ≈9.3 at 3 and 6 h, respectively; and zymosan in SGM also increased again at 6 h (SNR ≈ 9.0). These later signals may reflect cumulative intracellular processes, probe/medium chemistry or adaptive responses, and they should not automatically be interpreted as the initial oxidative insult. The practical implication is that a standardized early QC readout can coexist with repeated multi-time-point analysis of field samples.

The repeated-readout design therefore addresses a central limitation of single-endpoint measurements: an individual PM sample may exhibit an earlier or later maximum. DCFH-DA remains a broad redox-sensitive probe, so the kinetic profile is interpreted as a change in integrated cellular oxidative response, rather than as the time course of a specific ROS species [37,38].

Taken together, the present workflow differs incrementally from earlier cellular PM methods through the combined use of a human epithelial model, a PM-relevant reference dust, extended kinetic characterization, filter-substrate evaluation, and dual normalization. These features are summarized in Table 2. They constitute method-performance characterization, rather than proof of superiority to a universal gold-standard method, which does not currently exist for PM oxidative potential [39].

### 3.2. SRM 2584 Concentration–Response Results

**Figure 4:**
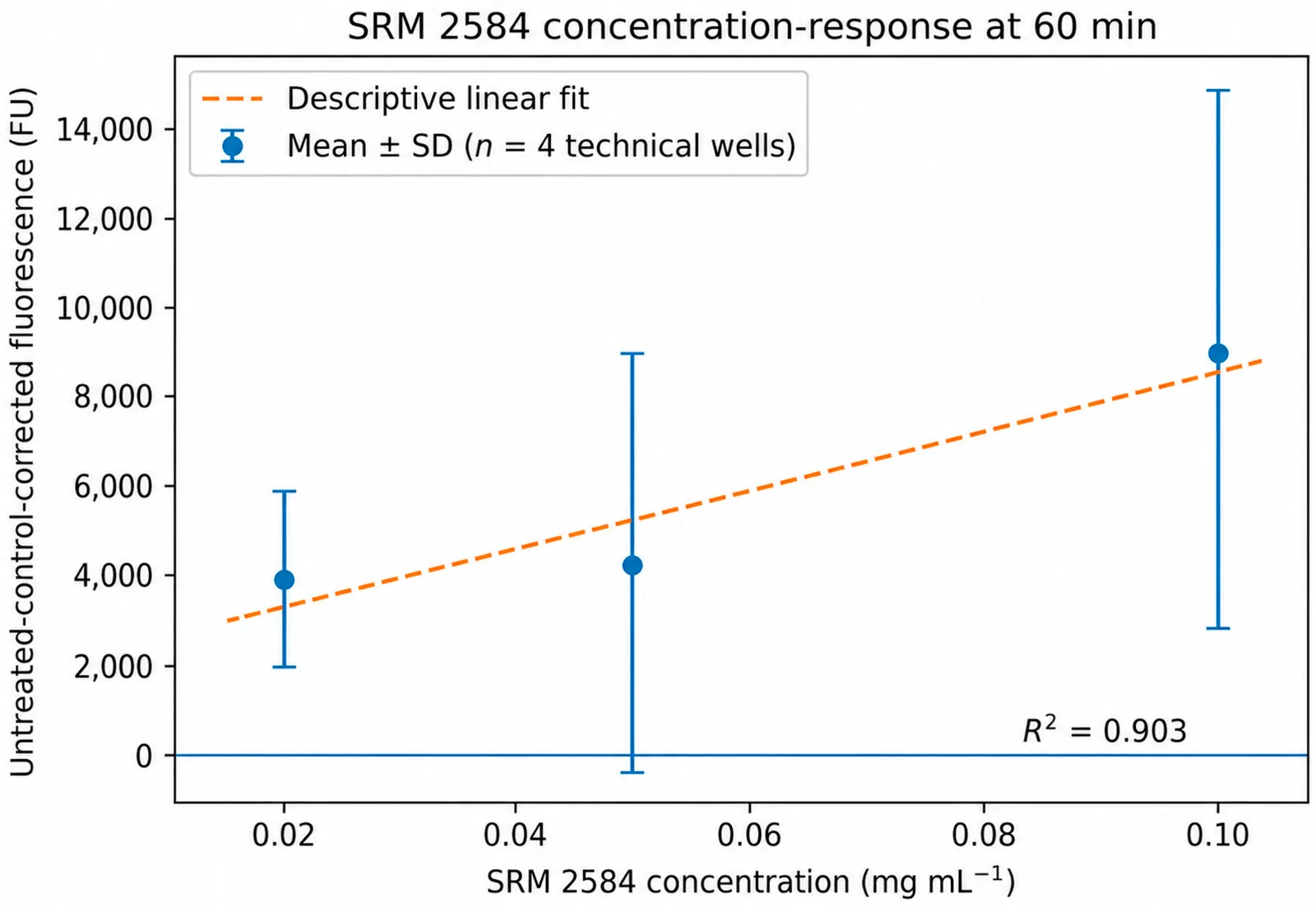
SRM 2584 concentration response at 60 min. Points show untreated-control-corrected mean fluorescence for 0.02, 0.05 and 0.10 mg mL^−1^; error bars show SD among *n* = 4 technical wells after subtraction of the common untreated-control mean. The dashed line is a descriptive linear fit across the three tested concentrations (*R*^2^ = 0.903). The concentration series was conducted in one dedicated independent experiment; no inferential significance is assigned, and the fitted interval should not be interpreted as a validated analytical linear range or upper detection limit.

The additional SRM 2584 concentration series showed that concentration–response behaviour depended on readout time. At 15, 30 and 45 min, the intermediate 0.05 mg mL^−1^ condition did not produce a monotonic response relative to 0.02 and 0.10 mg mL^−1^, indicating that a simple linear concentration–response relationship cannot be assumed at these early readouts. At 60 min, however, the untreated-control-corrected mean fluorescence increased from 3906.5 FU at 0.02 mg mL^−1^ to 4250.3 FU at 0.05 mg mL^−1^ and 8781.8 FU at 0.10 mg mL^−1^. A descriptive linear regression across the three tested concentrations at 60 min gave *R*^2^ = 0.903 (slope 6.40 × 10^4^ FU per mg mL^−1^; intercept 2021 FU). No plateau was evident at 0.10 mg mL^−1^ within the 60 min readout (Figure 4).

Because this concentration series experiment comprised one independent run with four technical wells per dose, and because the response was non-monotonic at earlier readouts, the 0.02–0.10 mg mL^−1^ interval is reported as an empirically tested concentration window, rather than as a validated analytical linear range. The data, nevertheless, demonstrate a concentration dependent increase at 60 min and reinforce the broader finding that interpretation of the cellular DCFH-DA response is readout-time dependent.

### 3.3. Influence of Filter Material on ROS Measurements (PTFE vs. Quartz)

Filter material is a potential methodological variable in PM characterization studies, because commonly used sampling substrates may differ in background signal, extraction behaviour, particle retention, compatibility with downstream chemical analysis, and possible interaction with the DCFH-DA-based fluorescence readout. Therefore, before applying the method to environmental PM samples, we specifically tested whether the type of filter itself could influence the ROS response measured in A549 cells. Two representative filter substrates widely used in PM sampling and characterization were selected for this comparison: PTFE (Teflon) filters, commonly used for gravimetric PM mass determination and elemental analysis, and quartz filters, commonly used when the determination of carbonaceous aerosol fractions such as OC/EC is required.

To examine the effect of filter substrate, PTFE and quartz filters were processed using the same extraction and assay workflow, and the corresponding blank responses were subtracted before comparison. The comparison used eight matched PTFE–quartz pairs. Maximum blank-corrected responses were compared on both particle-mass- and sampled-air-volume-normalized bases. To evaluate proportional agreement after blank correction, the regression was constrained through the origin. The resulting slopes were close to unity for both normalizations, demonstrating strong proportional agreement within this eight-pair comparison set. Given the limited number of paired samples, this finding is interpreted as method-comparison evidence within the tested workflow, rather than as proof of universal interchangeability between PTFE and quartz substrates.

**Figure 5:**
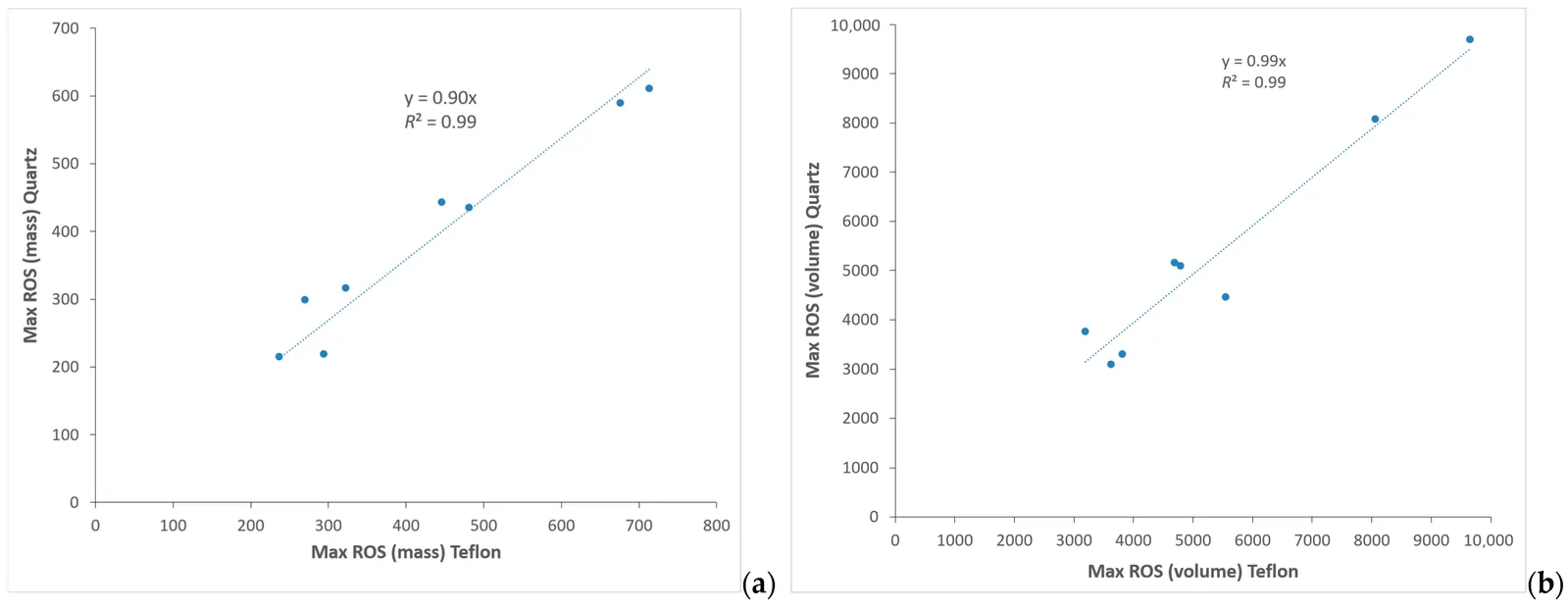
Linear regression analysis of maximum blank-corrected ROS fluorescence for the eight matched PTFE–quartz pairs retained in the finalized comparison: (**a**) mass-normalized response and (**b**) air-volume-normalized response. Linear fits were constrained through the origin; Blue dots represent the individual matched PTFE–quartz filter pairs, and the dashed line represents the corresponding zero-intercept linear regression fit.

For the eight matched pairs used for the comparison, zero-intercept regression yielded a slope of 0.897 with *R*^2^ = 0.994 for the mass-normalized responses and a slope of 0.985 with *R*^2^ = 0.991 for the air-volume-normalized responses (Figure 5).

**Figure 6:**
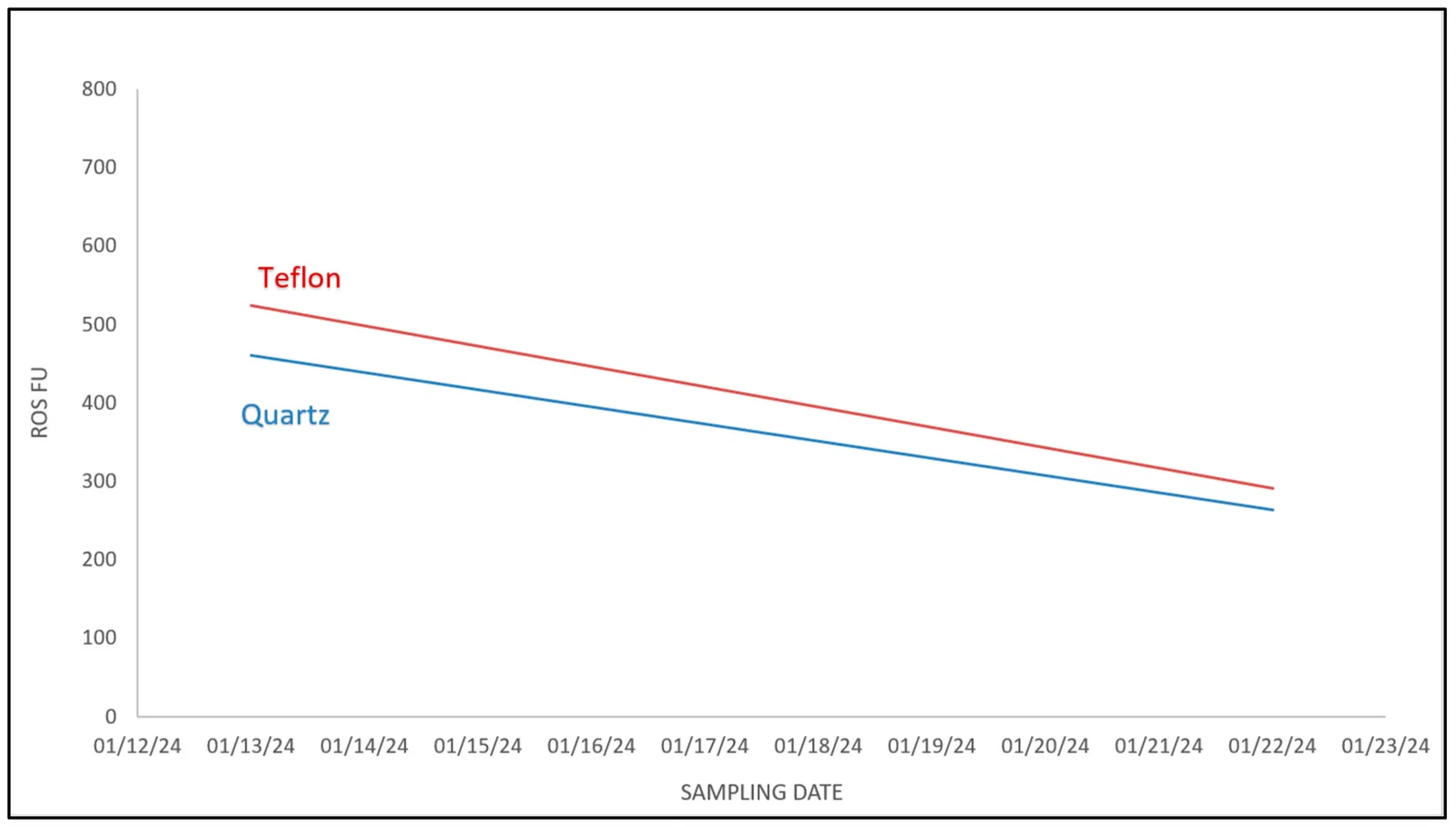
Illustrative comparison of maximum ROS fluorescence for selected PTFE and quartz filters.

These results indicate strong proportional agreement within the evaluated comparison set. Under the tested blank-correction and normalization workflow, no substantial proportional substrate-related difference was evident between the paired PTFE and quartz responses. This supports application of the assay to samples collected on either substrate under the tested conditions, while the limited eight-pair dataset does not establish universal interchangeability between PTFE and quartz filters (Figure 6).

### 3.4. ROS Activity of Field PM2.5 Samples Across Multiple Readout Times

Following the paired PTFE–quartz comparison, which showed strong proportional agreement within the eight-pair dataset, the characterized cellular assay was applied to field-collected filters from the DEM and (HAC)^2^ monitoring sites. The field measurements are presented first as blank-corrected fluorescence responses across multiple readout times; their relationship with PM loading and chemical composition is then discussed separately. as an exploratory analysis.

Following the experimental design and normalization procedure described in Section 2, the three field-filter series were examined in terms of blank-corrected fluorescence. Sample metadata for the PTFE filters collected at DEM and the quartz filters collected at (HAC)^2^ are summarized in Table 3 and Table 4, respectively.

For the first DEM series (2013), Figure 7a,b show blank-corrected fluorescence normalized to collected PM2.5 mass and sampled air volume, respectively, at 15, 30, 45 and 60 min. For this series, the larger responses were generally observed at 30–45 min. For the second DEM series (2019–2020), the corresponding repeated-readout profiles extend through 3 and 6 h (Figure 8a,b).

**Figure 7:**
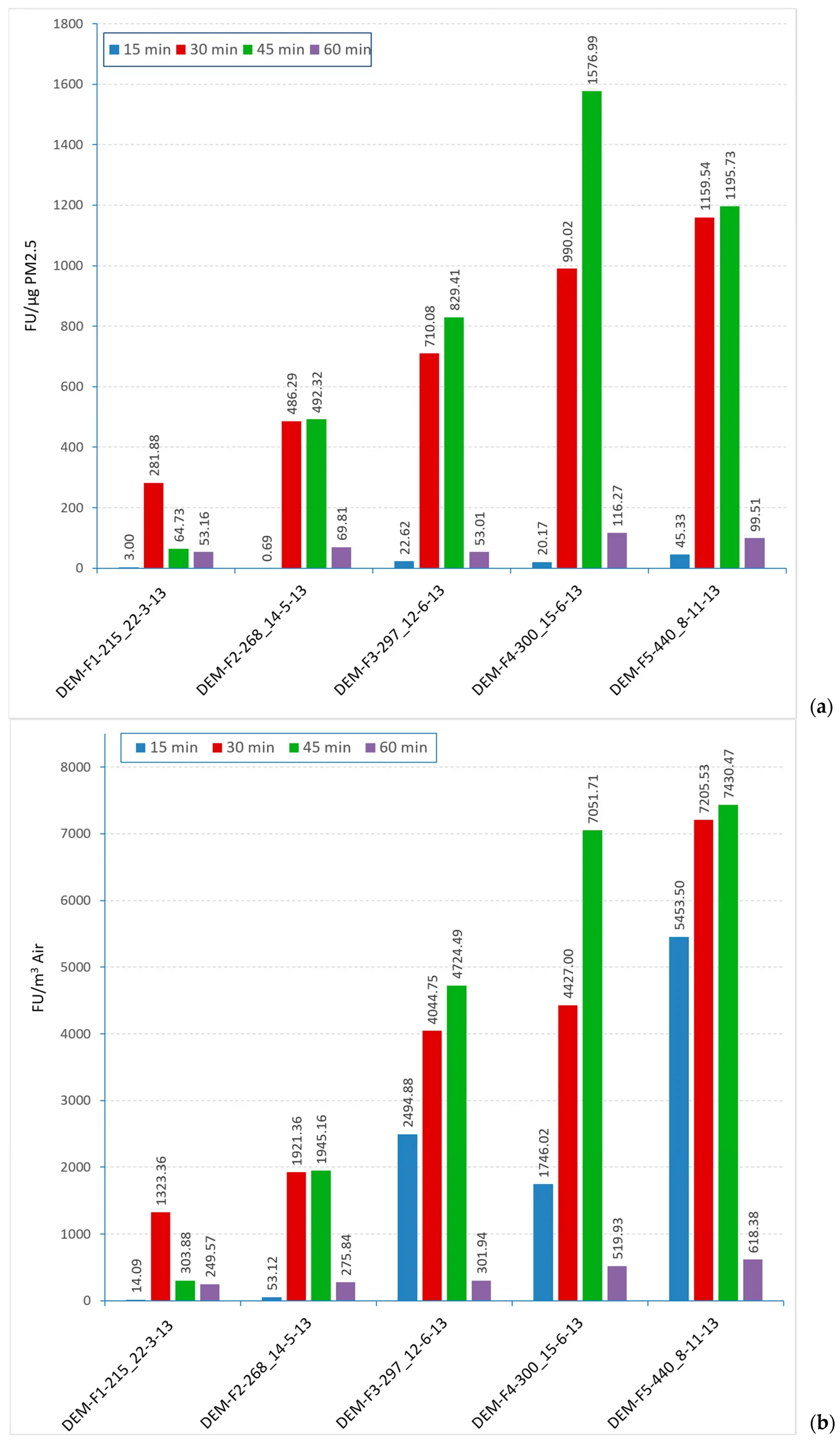
Blank-corrected fluorescence for the 2013 DEM filters expressed (**a**) per μg PM2.5 and (**b**) per m^3^ sampled air at 15, 30, 45 and 60 min. Bars show the arithmetic mean of *n* = 4 technical replicate wells for the corresponding filter extract and readout time. The environmental filters are distinct samples, rather than replicated treatment groups; no between-filter inferential significance testing was performed.

Comparison of Table 3 with Figure 7 indicates that PM mass concentration alone did not consistently explain the variation in cellular fluorescence among the 2013 DEM samples. For example, samples with similar PM2.5 mass showed markedly different mass-normalized responses, particularly at 30–45 min. This observation motivates examination of chemical composition, but it does not by itself establish which constituents caused the response. Air-volume-normalized fluorescence was higher for some June and November samples, indicating a greater oxidative-response burden per sampled air volume during those collection periods.

The field-filter results further illustrate why repeated readouts were retained. Maximum fluorescence was not observed at a common time across all samples: some maxima occurred at early readouts, whereas others appeared at 45 min or 3 h. A single endpoint could therefore miss the highest observed response for a given sample. These differences may reflect a combination of particle composition, uptake, cellular signalling, probe chemistry and secondary intracellular processes; the present dataset cannot isolate these mechanisms. The observed kinetic variability is considered below, in relation to PM chemical composition.

The quartz-filter series from Helmos is summarized in Table 4. These samples were used as the third field-filter application of the method and are discussed below in relation to their PM2.5 loading, ROS response and chemical composition.

For the (HAC)^2^ series, Table 4 reports the equivalent sampled air volume represented by the analysed filter portion used in the ROS assay, consistent with the air-volume normalization applied to that portion. PM2.5 mass concentration was calculated from the full filter loading and the corresponding total sampler air volume. Across the selected periods, the PM2.5 concentrations reported for (HAC)^2^ were generally lower than those for DEM; however, the sites, sampling configurations and sampling years were not matched, so the comparison is descriptive, rather than a controlled site-to-site contrast.

For the (HAC)^2^ quartz-filter series, Figure 9a,b present blank-corrected fluorescence normalized to PM2.5 mass and sampled air volume, respectively, at 15, 30, 45 and 60 min.

Figure 9 shows that the August 2016 (HAC)^2^ samples generally produced higher mass- and air-volume-normalized fluorescence than the February 2017 samples. The August samples also had higher PM loading. Because both PM mass and composition changed between the sampling periods, the present data support an association with overall sample characteristics, rather than attribution to a single determinant.

### 3.5. Chemical Composition and Exploratory Associations with ROS Activity

Because PM mass alone did not consistently explain the variation in fluorescence, chemical characterization was examined as a potential explanatory dimension. Major and trace elements were measured by XRF, and organic carbon (OC) and elemental carbon (EC) were determined by thermo-optical transmittance, as described in Section 2.3. The following comparisons are exploratory because the constituents occur as correlated mixtures and the number of paired chemistry-ROS observations is limited.

Table 5 consolidates the sampling period, filter substrate, PM2.5 concentration, key chemical parameters available for interpretation, and fluorescence-readout window for the field samples included in the ROS application. Detailed sample-level constituent profiles are shown in Figure 10, Figure 11, Figure 12 and Figure 13 and Supplementary Figures S1 and S2; Supplementary Tables S1 and S2 provide descriptive chemical statistics, while Tables S3 and S4 provide Fe/Cu–fluorescence comparisons for the 2013 DEM subset.

The elemental composition of the DEM 2013 samples is shown in Figure 10a and the corresponding OC/EC data in Figure 10b; Figure 11 presents the 2019–2020 DEM composition. Sulfur was among the more abundant measured elements in several samples, and coincided with high fluorescence for selected dates. However, XRF-derived sulfur does not identify the relevant sulfur chemical form, and sulfur-rich aerosol can covary with secondary inorganic aerosol, organic aerosol, acidity and metal solubility. The observed co-variation is therefore not interpreted as evidence that sulfur itself was the principal causal species. The relatively elevated Si, Ca and Fe levels are consistent with mineral/construction-related contributions, while the full elemental and carbonaceous data are provided in Figure S1 and Table S1.

The elevated Al and Si observed on 12 June 2013 are consistent with a possible mineral-dust contribution, while the Na and Cl pattern on 22 March 2013 is consistent with sea-salt influence. Cu, Mn, Cr and Zn can be associated with non-exhaust traffic and other anthropogenic sources, and the high OC/EC ratios may reflect a mixture of combustion and secondary organic aerosol. These source indications are qualitative; event-specific source apportionment or back-trajectory evidence would be required for definitive attribution.

Figure 12 and Figure 13 summarize elemental and OC/EC composition for the (HAC)^2^ samples collected in August 2016 and February 2017, respectively; descriptive statistics are provided in Table S2. Sulfur was relatively abundant compared with several trace elements in these samples. At a remote high-altitude site, such sulfur may reflect regional or long-range transported secondary aerosol. The present measurements alone, however, cannot identify a specific refinery or other single source without trajectory/source-apportionment analysis.

**Figure 8:**
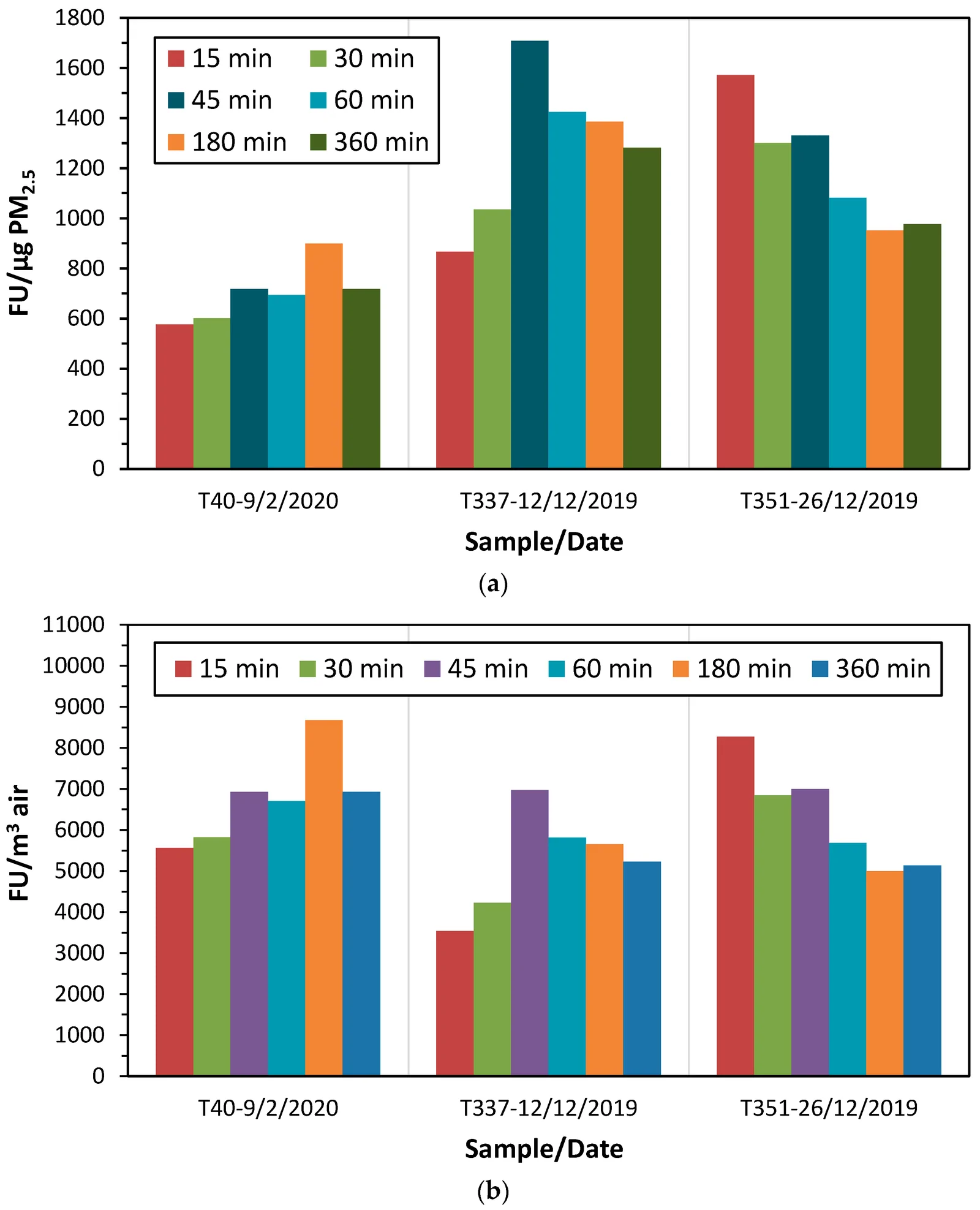
Blank-corrected fluorescence for representative 2019–2020 DEM filters expressed (**a**) per μg PM2.5 and (**b**) per m^3^ sampled air over the 15 min–6 h time course. Each bar represents the mean of *n* = 4 technical replicate wells for the corresponding filter extract and readout time. The environmental filters are distinct samples, rather than replicated treatment groups.

**Figure 9:**
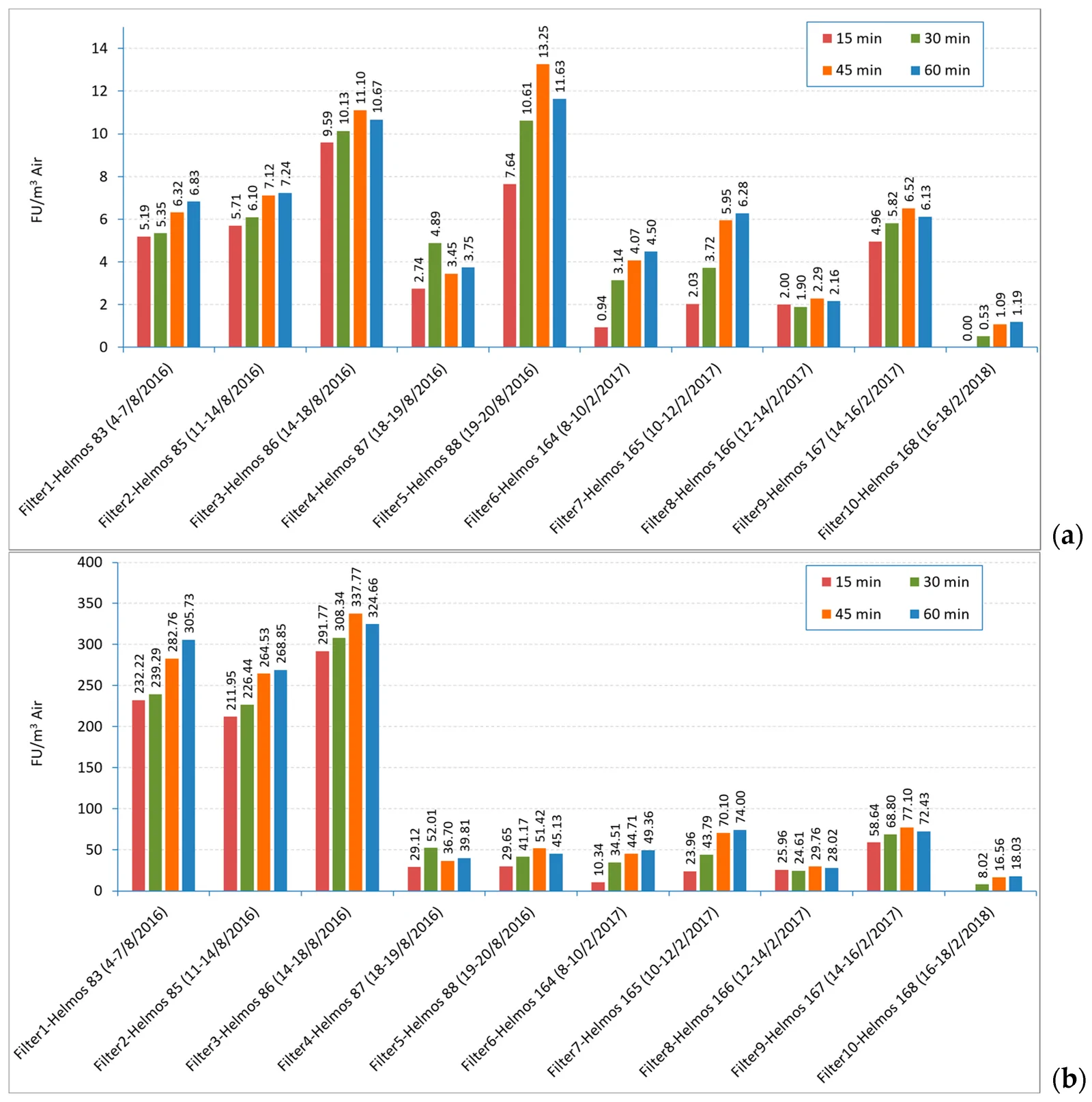
Blank-corrected fluorescence for the (HAC)^2^ quartz-filter series expressed (**a**) per μg PM2.5 and (**b**) per m^3^ sampled air at 15, 30, 45 and 60 min. Bars show the arithmetic mean of *n* = 4 technical replicate wells for the corresponding filter extract and readout time. The environmental filters are distinct samples, rather than replicated treatment groups; comparisons are descriptive and no between-filter inferential significance testing was performed.

**Figure 10:**
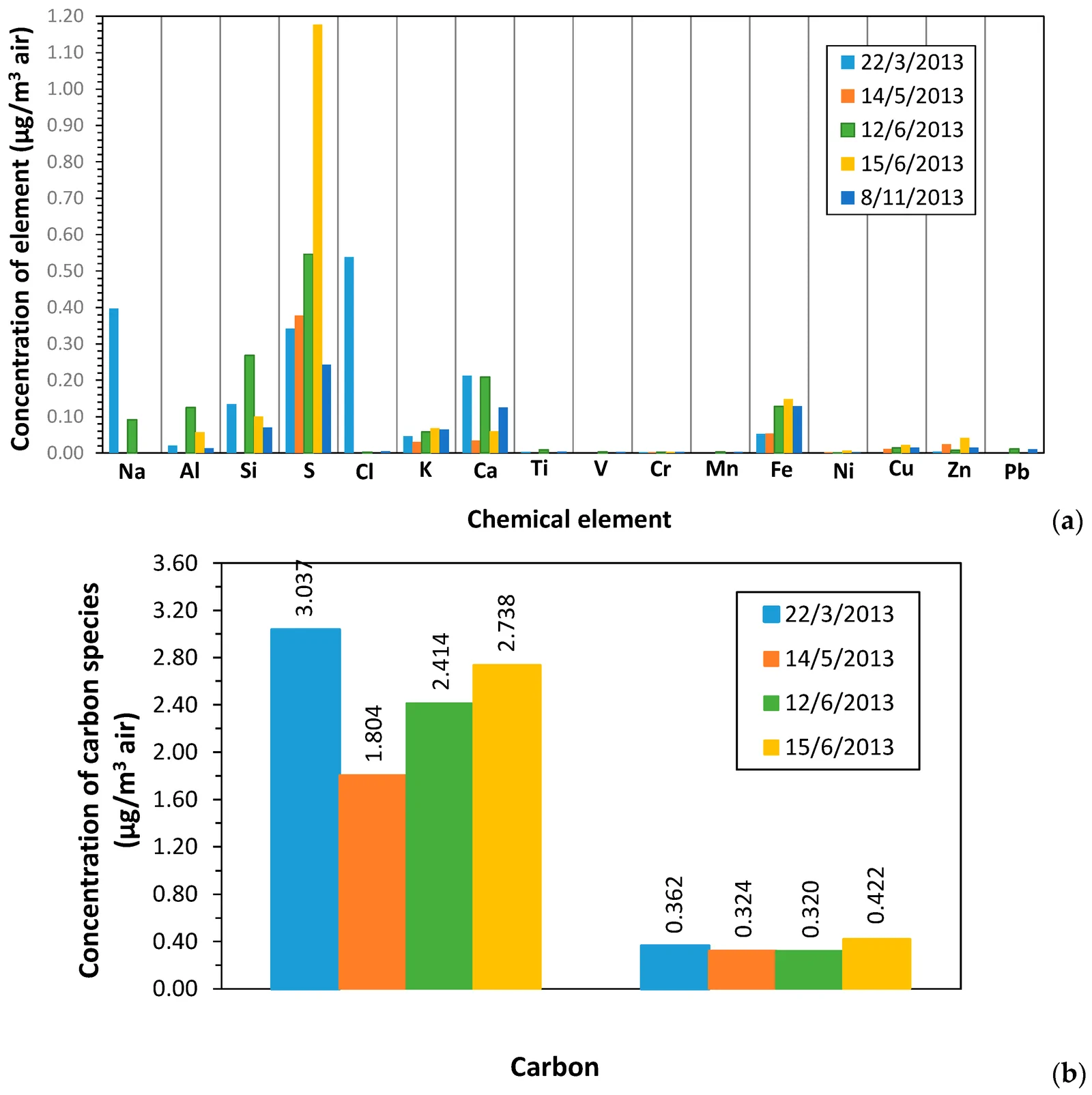
(**a**) Concentrations of the detected elements on the PM2.5 particles collected at the DEM station during **2013**; (**b**) concentrations of organic carbon (OC) and elemental carbon (EC) in the corresponding samples.

**Figure 11:**
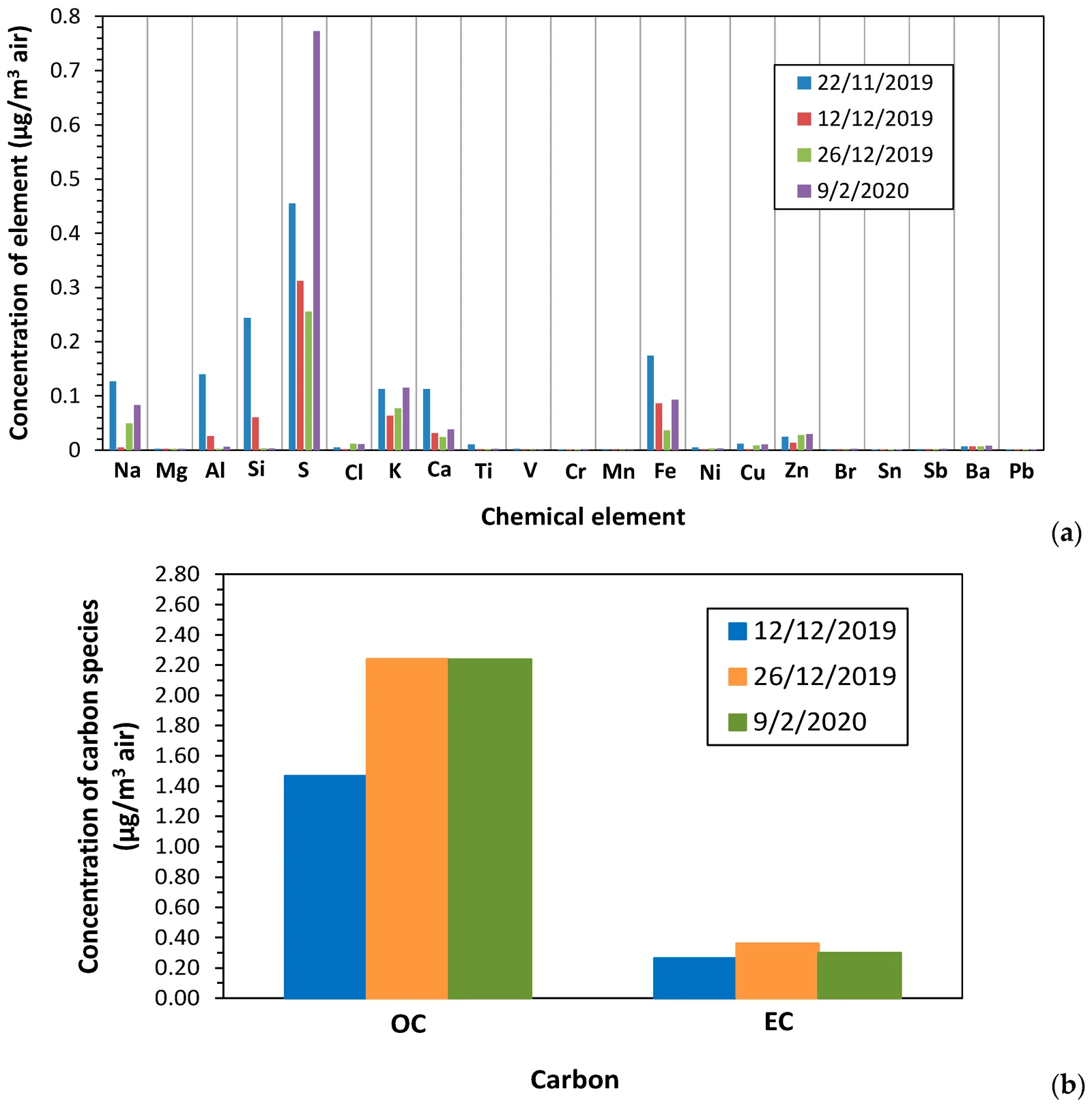
(**a**) Concentrations of the detected elements in PM2.5 collected at the DEM station during 2019–2020; (**b**) concentrations of organic carbon (OC) and elemental carbon (EC) in the corresponding samples.

**Figure 12:**
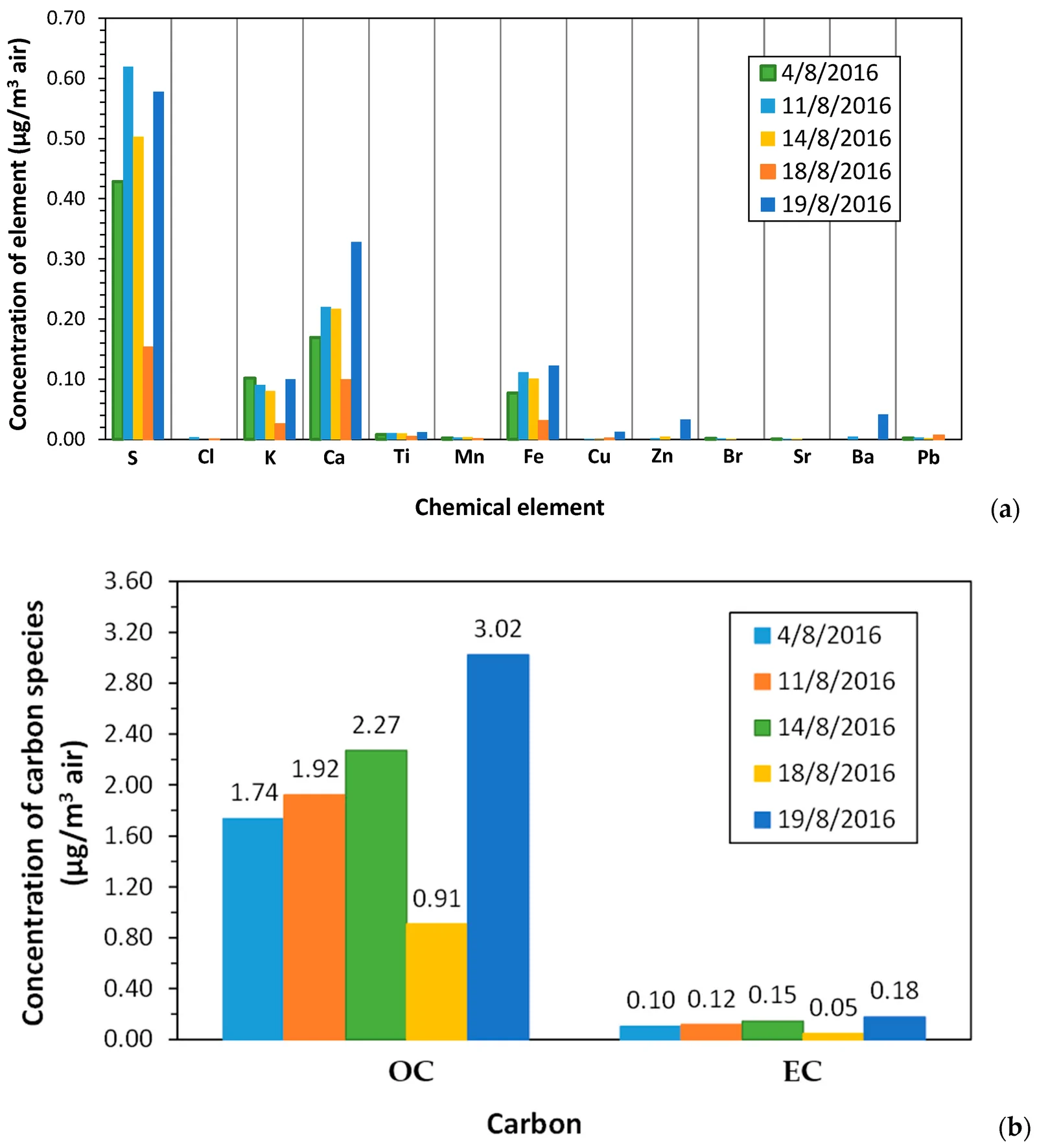
(**a**) Concentrations of the chemical elements on the PM2.5 particles adsorbed in the filters derived from the (HAC)^2^ station on mount Helmos, (**b**) concentrations of the OC and EC on the PM2.5 particles on the same filters. The filters were collected in August 2016.

**Figure 13:**
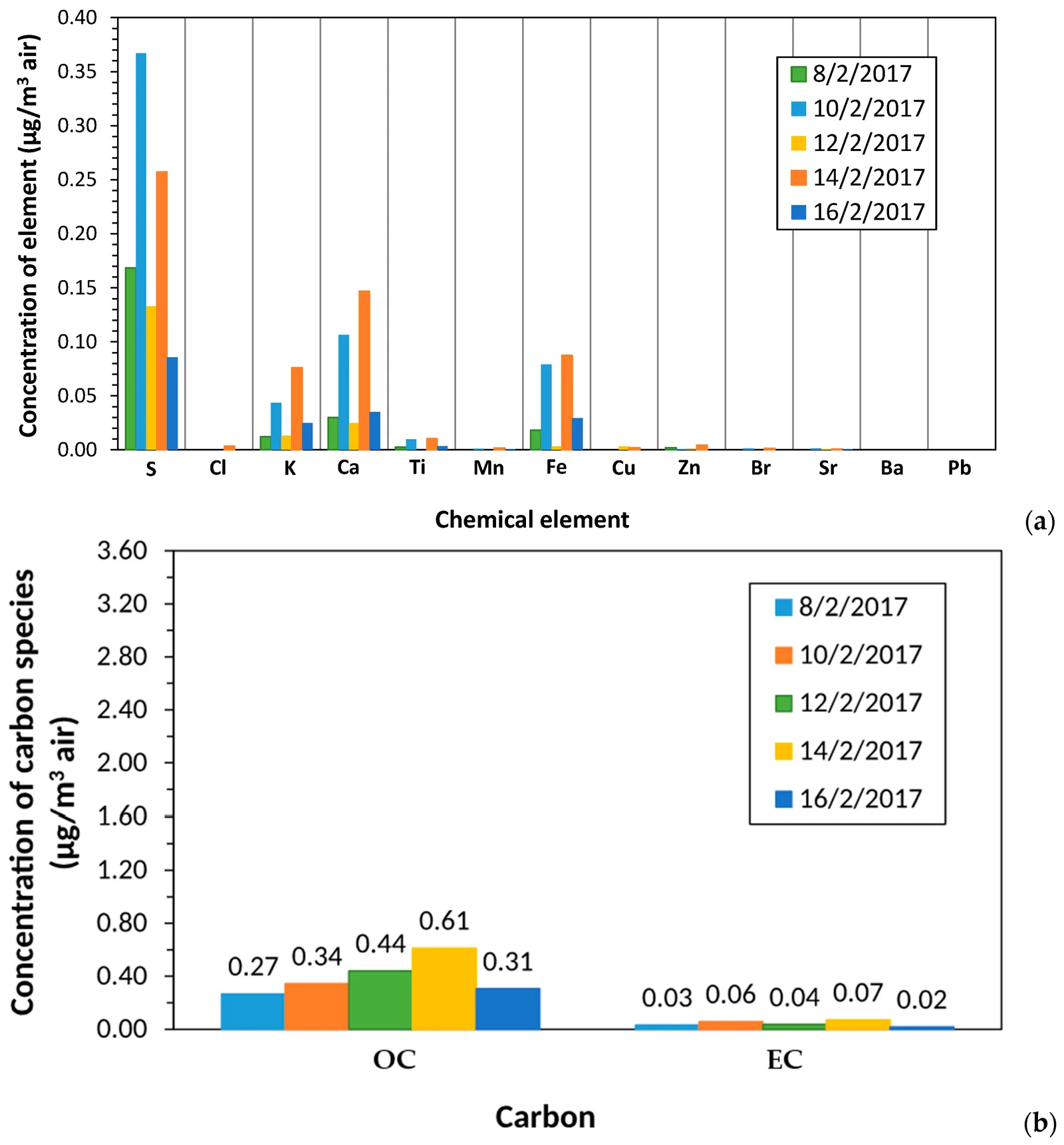
(**a**) Concentrations of the chemical elements on the PM2.5 particles adsorbed in the filters derived from the (HAC)^2^ station, (**b**) concentrations of the OC and EC on the PM2.5 particles in the same filters. The filters were collected in February 2017.

**Figure 14:**
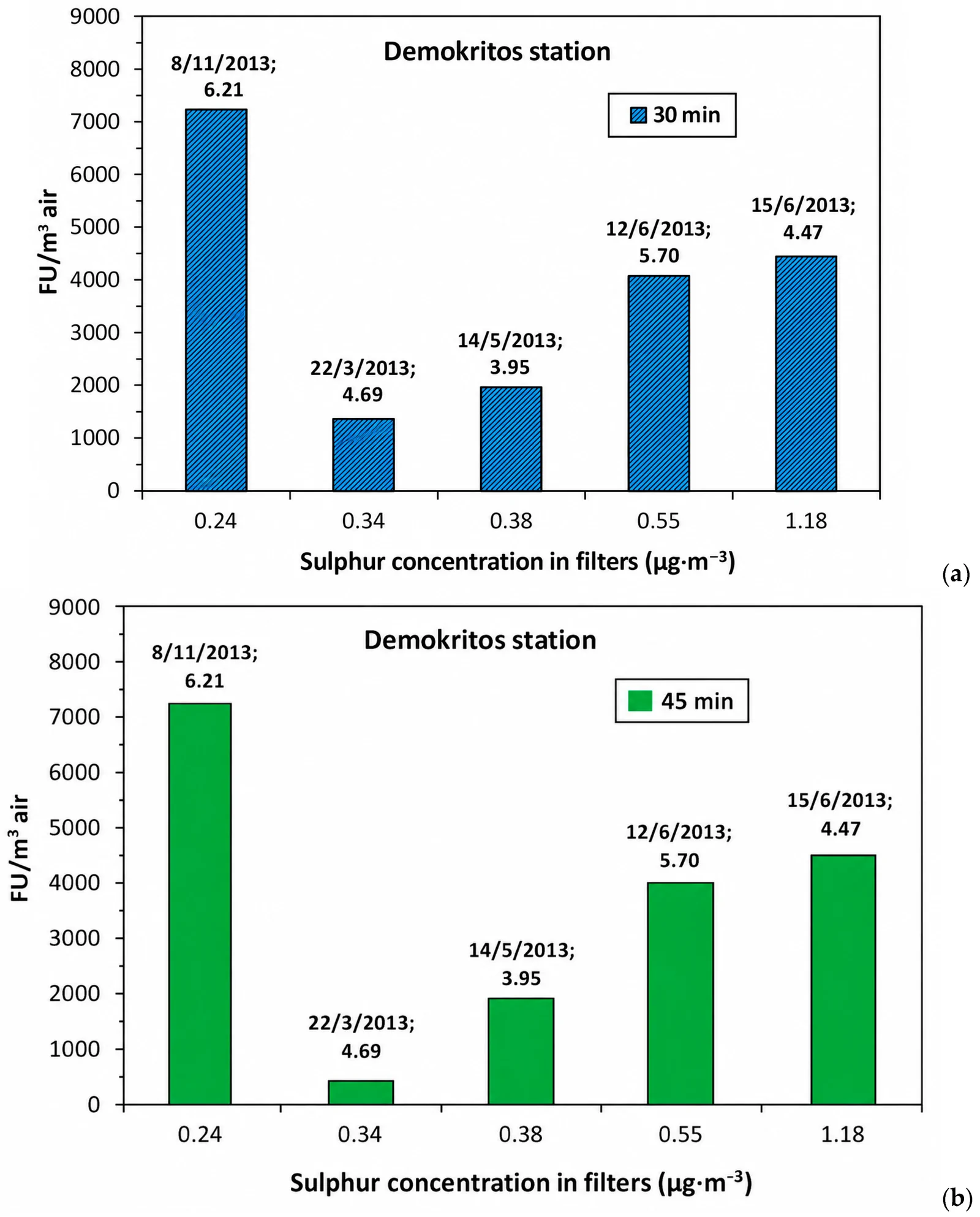
Exploratory comparison of air-volume-normalized fluorescence with sulfur concentration in the 2013 DEM filters at (**a**) 30 min and (**b**) 45 min. Labels above the bars indicate the corresponding PM2.5 concentration (μg m^−3^). The figure illustrates co-variation in a small set of chemically distinct samples, and is not a constituent-specific dose–response analysis.

Across the small set of chemically characterized filters, higher sulfur concentrations coincided with higher fluorescence for some DEM and (HAC)^2^ samples (Figure 7, Figure 8, Figure 9, Figure 10, Figure 11, Figure 12, Figure 13 and Figure 14). This pattern is best described as co-variation, rather than correlation, in the inferential sense, because the sample number is limited and sulfur covaries with other aerosol components. The data, therefore, do not demonstrate that sulfur compounds directly stimulated the observed A549 response, nor do they quantify an independent sulfur effect.

**Figure 15:**
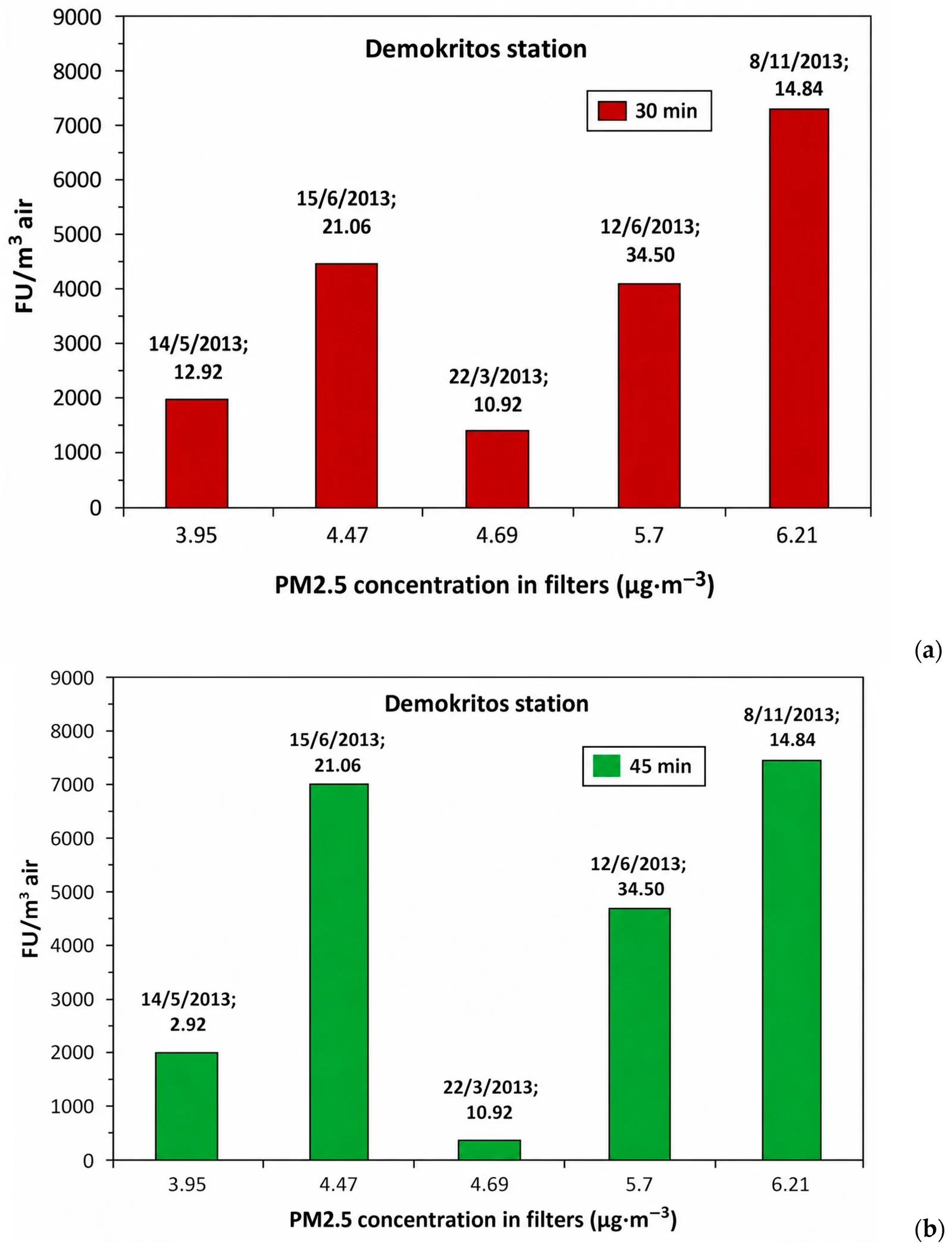
Exploratory comparison of air-volume-normalized fluorescence with PM2.5 concentration in the 2013 DEM filters at (**a**) 30 min and (**b**) 45 min. Labels above the bars indicate the corresponding total sulfur mass (μg). The figure is descriptive, and does not isolate independent effects of PM mass or sulfur.

Figure 14 and Figure 15 illustrate why neither sulfur concentration nor PM2.5 mass alone provides a complete explanation of the fluorescence response. For example, the sample with the highest PM2.5 concentration did not have the highest sulfur concentration, yet it produced the highest air-volume-normalized fluorescence. Conversely, some samples with increasing sulfur also showed increasing fluorescence. These patterns are consistent with a multicomponent aerosol effect, and should not be interpreted as monotonic dose–response evidence for sulfur.

The contrasting behaviour in Figure 15 further argues against assigning a dominant effect to either sulfur or bulk PM mass. The relative contributions of secondary inorganic species, carbonaceous material and redox-active metals cannot be separated from these descriptive comparisons. A larger dataset with ion/organic speciation and multivariable or source-apportionment analysis would be required to estimate independent component effects. A broader spatially and seasonally resolved sample set across contrasting atmospheric environments would be needed to test whether these exploratory constituent–response patterns persist across a wider range of PM mixtures.

K, Fe and Cu also showed some qualitative co-variation with fluorescence in the DEM series (Tables S3 and S4), while other measured constituents did not exhibit an obvious pattern in this small sample set. We avoid describing these observations as positive or negative correlations because no adequately powered constituent-specific correlation analysis was performed. In complex PM mixtures, transition metals and organic components may contribute jointly or interact, and the present field dataset is intended to generate hypotheses, rather than establish causal component–response relationships.

### 3.6. Study Limitations

Key scope considerations should be noted. A549 is a transformed human lung epithelial cell line, and DCFH-DA is a broad, non-species-selective redox-sensitive probe; accordingly, the results are interpreted as an integrated cellular oxidative-response signal, and should not be assumed to generalize directly to primary or multicellular pulmonary models without further testing [37,38,50]. The control strategy deliberately emphasized the PM-relevant particulate reference SRM 2584. A classical soluble oxidant and a parallel independent oxidative-potential assay were not included in the present study, but could provide complementary context in comparative work, particularly because PM oxidative-potential methods are method-dependent and are not harmonized to a single universal reference method [39]. Finally, the paired-filter and field chemistry–response analyses involve relatively limited sample sets, and therefore support proportional method comparison and exploratory co-variation, respectively, rather than universal substrate interchangeability or constituent-specific causal attribution. These considerations define the scope of the present method-performance characterization and field-application study. Broader evaluation using additional pulmonary models, complementary assay endpoints, larger and more diverse PM sample sets, and additional independent concentration-series experiments would be needed to examine transferability, constituent-specific effects and a formal analytical working range.

## 4. Conclusions

This study characterizes an A549 cell-based DCFH-DA approach using repeated fluorescence readouts for assessing the oxidative activity of filter-collected atmospheric PM2.5. The methodological contribution is the integration of a human epithelial model with assay-specific controls, a PM-relevant reference dust, an extended kinetic series, filter-substrate evaluation, and dual normalization by particle mass and sampled air volume.

Measurements across the extended kinetic series showed that the fluorescence response is stimulus-dependent. Within the early 15–60 min window, 30 min is useful as a standardized quality-control checkpoint for SRM 2584 and zymosan in SGM, but the extended data demonstrate later maxima for some conditions. Multiple readout times are therefore retained for environmental samples, rather than assuming a universal single endpoint. The added SRM 2584 concentration series further showed readout-dependent dose behaviour: the 60 min untreated-control-corrected response increased across 0.02–0.10 mg mL^−1^ and followed an approximate descriptive linear trend (*R*^2^ = 0.90), whereas the 15–45 min responses were non-monotonic. This supports use of SRM 2584 as a strong PM-relevant reference, while limiting any claim of a universal linear working range.

After substrate-specific blank correction and normalization, the eight matched PTFE–quartz pairs retained in the finalized regression analysis showed strong proportional agreement, with zero-intercept slopes close to unity for both particle-mass- and air-volume-normalized responses. No substantial proportional substrate-related difference was evident within this comparison set, although the limited number of pairs restricts generalization.

Application to field-collected PM2.5 samples demonstrated the practical applicability of the characterized workflow beyond the reference-control experiments. The cellular oxidative-response profiles differed among environmental samples, and did not track PM mass alone, supporting the use of the assay for investigating variations in PM-induced oxidative activity alongside particle chemical characterization. Differences in elemental and carbonaceous composition were associated with differences in the cellular response; however, the present field dataset and covariance among constituents do not support causal attribution to sulfur or any other single component.

Overall, the present study provides a characterized and field-applied methodological basis for assessing PM-induced cellular oxidative responses across multiple readout times. The field results show that PM mass alone does not capture the observed variability in oxidative-response profiles, while differences in particle composition coincide with differences in cellular response. These findings emphasize the value of integrating detailed chemical characterization with mass-based measurements when interpreting the toxicological relevance of atmospheric PM.

## Figures and Tables

**Table 1:** Overall CV and SNR (15–60 min).

Scenario	Medium	UT CV Mean (Range) %	POS CV Mean (Range) %	Mean SNR (Range)
Zym in PBS	PBS	13.0 (11.4–15.6)	4.7 (1.7–10.4)	1.05 (–0.71–2.10)
SRM 2584 in PBS	PBS	13.0 (11.4–15.6)	16.6 (10.5–25.9)	18.2 (10.95–23.20)
Zym in SGM	SGM	3.1 (0.68–6.11)	6.9 (3.93–9.79)	3.88 (1.66–6.40)

**Table 2:** Representative comparison of cellular PM oxidative-response method-evaluation approaches and the present study.

Approach	Cell Model/Probe	Positive or Reference Control	Timing/Performance Emphasis	Relevance to the Present Study
Landreman et al. [33]	NR8383 rat alveolar macrophages/DCFH-DA	Zymosan in SGM	Macrophage-based PM-ROS protocol; routine filter-sample monitoring	Foundational particulate cellular ROS workflow; SGM used as a simple exposure medium.
Salana et al. [52]	NR8383 macrophages/DCFH-DA	t-BOOH (t-BHP)	Semi-automated SCOPE method; precision/accuracy and manual-method comparison	Shows the role of a defined chemical oxidant as an assay-functionality control.
Fang et al. [32]	NR8383 macrophage system/DCFH-DA	Zymosan and reference dust	Interlaboratory reproducibility, SNR, concentration–response and normalization	Benchmark for CV/SNR characterization and PM-relevant reference controls.
Present study	Human A549 lung epithelial cells/DCFH-DA	NIST SRM 2584 in PBS; zymosan comparator; assay-specific controls	15 min–6 h repeated-readout kinetic characterization; three-concentration SRM 2584 series (0.02–0.10 mg mL^−1^); PTFE/quartz paired comparison (*n* = 8); dual normalization	Extends the workflow to a human epithelial model, explicitly evaluates time dependence, and treats filter-substrate effects as a source of methodological uncertainty. A chemically defined oxidant was not included.

**Table 3:** Sample codes, filter exposure dates, volume of supplied air, total masses and concentrations of PMs in the filters collected from the DEM station.

Filter Coding	Start Date	Supplied Air Volume at Ambient Conditions (m^3^)	Total Mass of PMs on Filter (mg)	Concentration of PMs at Ambient Conditions (μg/m^3^)
DEM2013T215	22 March 2013	31.95	0.15	4.69
DEM2013T268	14 May 2013	34.17	0.135	3.95
DEM2013T297	12 June 2013	63.20	0.36	5.70
DEM2013T300	15–16 June 2013	17.89	0.08	4.47
DEM2013T440	8–9 November 2013	61.15	0.38	6.21
DEM2019T337	12 December 2019	55.14	0.23	4.08
DEM2019T351	26 December 2019	55.13	0.29	5.26
DEM2020T40	9 February 2020	55.12	0.53	9.66

**Table 4:** Sample codes, filter exposure dates, equivalent analysed air volume, total PM mass, and PM2.5 concentration for the (HAC)^2^ filters.

Filter Coding	Sampling Period	Analysed Air-Volume Equivalent (m^3^)	Total Mass of PMs on Filter (mg)	Concentration of PMs at Ambient Conditions (μg/m^3^)
Filter1-Helmos 83	4–7 August 2016	119.22	5.336	3.229
Filter2-Helmos 85	11–14 August 2016	117.39	4.360	2.680
Filter3-Helmos 86	14–18 August 2016	117.85	3.586	2.195
Filter4-Helmos 87	18–19 August 2016	117.72	1.251	0.767
Filter5-Helmos 88	19–20 August 2016	117.36	0.455	0.280
Filter6-Helmos 164	8–10 February 2017	118.03	1.296	0.792
Filter7-Helmos 165	10–12 February 2017	117.73	1.387	0.850
Filter8-Helmos 166	12–14 February 2017	117.99	1.532	0.937
Filter9-Helmos 167	14–16 February 2017	117.28	1.387	0.853
Filter10-Helmos 168	16–18 February 2017	117.12	1.780	1.096

**Table 5:** Sample-level overview of the field PM2.5 samples and key analytical parameters used in the composition–response assessment.

Sample	Site/Substrate	Sampling Period	PM2.5 (μg m^−3^)	Key Chemistry/Data Location	Fluorescence Readouts
DEM2013T215	DEM/PTFE	22 March 2013	4.69	XRF (incl. S, Fe, Cu) + OC/EC; Figure 10 and Figure S1	15, 30, 45, 60 min
DEM2013T268	DEM/PTFE	14 May 2013	3.95	XRF (incl. S, Fe, Cu) + OC/EC; Figure 10 and Figure S1	15, 30, 45, 60 min
DEM2013T297	DEM/PTFE	12 June 2013	5.70	XRF (incl. S, Fe, Cu) + OC/EC; Figure 10 and Figure S1	15, 30, 45, 60 min
DEM2013T300	DEM/PTFE	15–16 June 2013	4.47	XRF (incl. S, Fe, Cu) + OC/EC; Figure 10 and Figure S1	15, 30, 45, 60 min
DEM2013T440	DEM/PTFE	8–9 November 2013	6.21	XRF (incl. S, Fe, Cu); Figure 10 and Figure S1	15, 30, 45, 60 min
DEM2019T337	DEM/PTFE	12 December 2019	4.08	XRF (incl. S, Fe, Cu) + OC/EC; Figure 11	15, 30, 45, 60 min, 3 h, 6 h
DEM2019T351	DEM/PTFE	26 December 2019	5.26	XRF (incl. S, Fe, Cu) + OC/EC; Figure 11	15, 30, 45, 60 min, 3 h, 6 h
DEM2020T40	DEM/PTFE	9 February 2020	9.66	XRF (incl. S, Fe, Cu) + OC/EC; Figure 11	15, 30, 45, 60 min, 3 h, 6 h
Filter1-Helmos 83	(HAC)^2^/quartz	4–7 August 2016	3.229	XRF (incl. S, Fe, Cu) + OC/EC; Figure 12 and Figure S2	15, 30, 45, 60 min
Filter2-Helmos 85	(HAC)^2^/quartz	11–14 August 2016	2.680	XRF (incl. S, Fe, Cu) + OC/EC; Figure 12 and Figure S2	15, 30, 45, 60 min
Filter3-Helmos 86	(HAC)^2^/quartz	14–18 August 2016	2.195	XRF (incl. S, Fe, Cu) + OC/EC; Figure 12 and Figure S2	15, 30, 45, 60 min
Filter4-Helmos 87	(HAC)^2^/quartz	18–19 August 2016	0.767	XRF (incl. S, Fe, Cu) + OC/EC; Figure 12 and Figure S2	15, 30, 45, 60 min
Filter5-Helmos 88	(HAC)^2^/quartz	19–20 August 2016	0.280	XRF (incl. S, Fe, Cu) + OC/EC; Figure 12 and Figure S2	15, 30, 45, 60 min
Filter6-Helmos 164	(HAC)^2^/quartz	8–10 February 2017	0.792	XRF (incl. S, Fe, Cu) + OC/EC; Figure 13 and Figure S2	15, 30, 45, 60 min
Filter7-Helmos 165	(HAC)^2^/quartz	10–12 February 2017	0.850	XRF (incl. S, Fe, Cu) + OC/EC; Figure 13 and Figure S2	15, 30, 45, 60 min
Filter8-Helmos 166	(HAC)^2^/quartz	12–14 February 2017	0.937	XRF (incl. S, Fe, Cu) + OC/EC; Figure 13 and Figure S2	15, 30, 45, 60 min
Filter9-Helmos 167	(HAC)^2^/quartz	14–16 February 2017	0.853	XRF (incl. S, Fe, Cu) + OC/EC; Figure 13 and Figure S2	15, 30, 45, 60 min
Filter10-Helmos 168	(HAC)^2^/quartz	16–18 February 2017	1.096	XRF (incl. S, Fe, Cu) + OC/EC; Figure 13 and Figure S2	15, 30, 45, 60 min

## Data Availability

The data presented in this study are available within the article and its Supplementary Materials. Additional raw data supporting the findings of this study, including the fluorescence and chemical-composition datasets, are available from the corresponding author upon reasonable request.

## Supplementary Materials

The following supporting information can be downloaded at: https://www.mdpi.com/article/10.3390/toxics14090789/s1, Figure S1: Concentrations of the detected chemical elements, organic carbon, and elemental carbon, which were adsorbed on the filters collected from the station at NCSRD throughout 2013; Figure S2: Concentrations of the detected chemical elements, organic carbon, elemental carbon, and PM2.5, which were adsorbed on the filters collected from the station on mount Helmos during August 2016 and February 2017; Table S1: Statistical analysis of XRF measurements on the filters collected from the NCSRD station throughout the year 2013; Table S2: Statistical analysis of XRF measurements on the filters collected from the mount Helmos station during 8/2016 and 2/2017; Table S3: Variation of the supplied air volume, concentration and total mass of PM2.5 and Fe, and fluorescence intensity per m^3^ air, for the time intervals of 30 min and 45 min; Table S4: Variation of the supplied air volume, concentration and total mass of PM_2.5_ and Cu, and fluorescence intensity per m^3^ air, for the time intervals of 30 min and 45 min.

## Abbreviations

The following abbreviations are used in this manuscript:

A549	Human lung adenocarcinoma epithelial cell line A549
CAA	Cellular Antioxidant Activity
CV	Coefficient of variation
DCFH-DA	2′,7′-Dichlorodihydrofluorescein diacetate
DEM	Demokritos Atmospheric Aerosol Measurement station
DNA	Deoxyribonucleic acid
EC	Elemental carbon
ED-XRF	Energy-dispersive X-ray fluorescence
EDTA	Ethylenediaminetetraacetic acid
EUSAAR2	European Supersites for Atmospheric Aerosol Research, protocol 2
FU	Fluorescence unit(s)
(HAC)^2^	Helmos Hellenic Atmospheric Aerosols and Climate Change Station
NCSRD	National Centre for Scientific Research “Demokritos”
NIST	National Institute of Standards and Technology
NR8383	Rat alveolar macrophage cell line NR8383
OC	Organic carbon
OC/EC	Organic-carbon-to-elemental-carbon ratio
OP	Oxidative Potential
PBS	Phosphate-buffered saline
PM	Particulate matter
PM_2.5_	Particulate matter with an aerodynamic diameter of 2.5 μm or less
PM_10_	Particulate matter with an aerodynamic diameter of 10 μm or less
POS	Positive control
PT	Positive control
PTFE	Polytetrafluoroethylene
ROS	Reactive oxygen species
SD	Standard deviation
SGM	Salt–glucose medium
SNR	Signal-to-noise ratio
SRM	Standard Reference Material
UT	Untreated control
WHO	World Health Organization
XRF	X-ray fluorescence
Zym	Zymosan
