# Supplementary material for: An A549 Cell-Based Approach Using Repeated Fluorescence Readouts for Assessing Reactive Oxygen Species Activity of Atmospheric Particulate Matter
Source: Toxics. 2026 Sep 7;14(9):789. doi: 10.3390/toxics14090789 (PMC13611699; doi:10.3390/toxics14090789)
Supplement: Supplementary file 1 [file toxics-14-00789-s001.zip › toxics-4483200-supplementary.pdf]

Supplementary materials

An improved in-vitro approach for the assessment of Reactive Oxygen Species activity of atmospheric Particulate Matter on human cells

Ioanna Tzagkaroulaki[1,3,*], Evangelia Diapouli[1,*], Vasiliki Vasilatou [1], Stefanos Papagiannis[1,4] and Efthimios Tagaris[3]

[1] National Centre for Scientific Research 'Demokritos', 15341 Agia Paraskevi, Athens, Greece
[2] University of Western Macedonia, Department of Mechanical Engineering, ZEP, 50150 Kozani, Greece
[3] University of Western Macedonia, Department of Chemical Engineering, ZEP, 50150 Kozani, Greece
[4] University of Ioannina, 45110, Ioannina, Greece
* Correspondence: ioanna@ipta.demokritos.gr; Tel.: +302106503742 (IT); ldiapouli@ipta.demokritos.gr; Tel.: +302106503259 (ED)

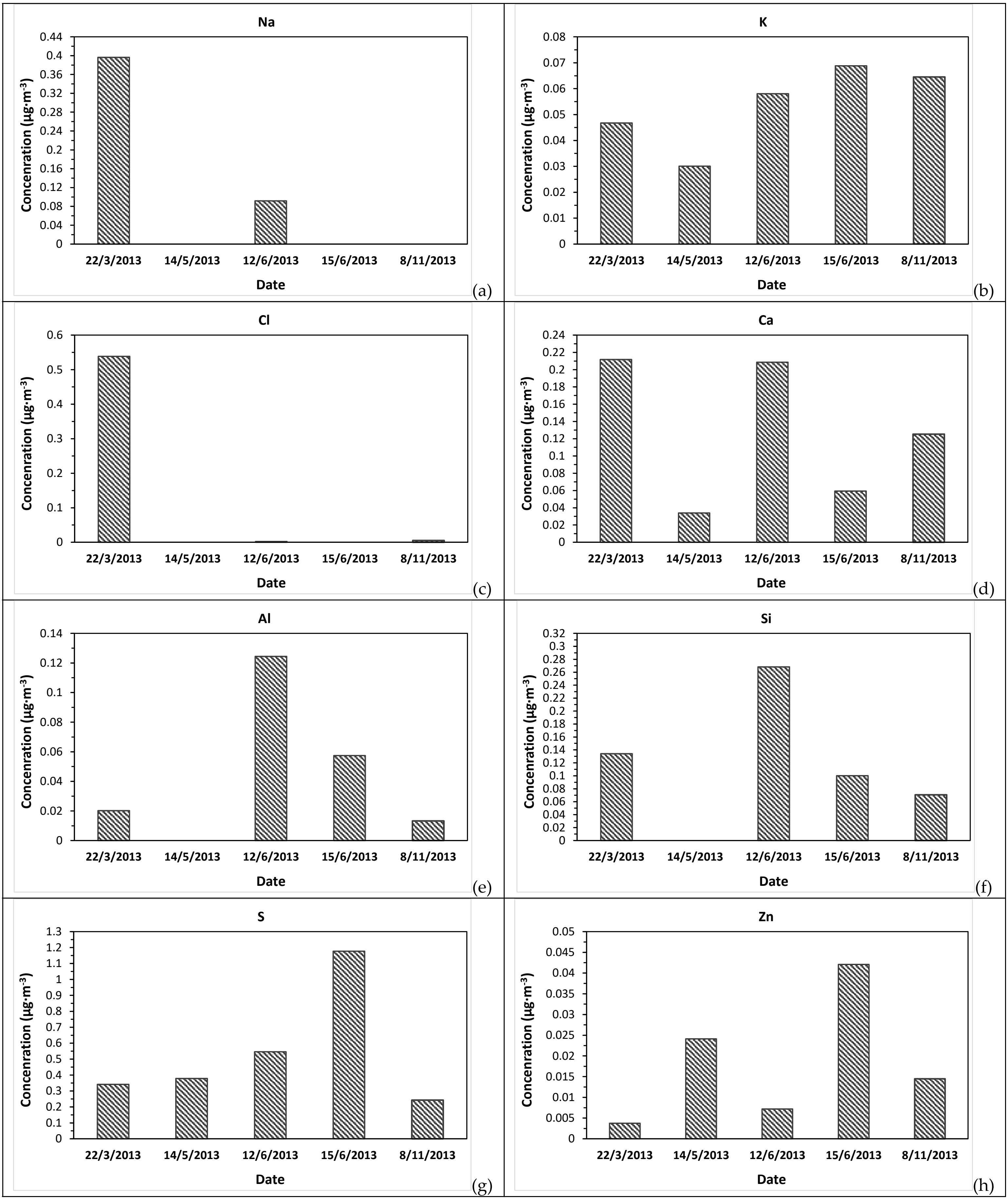
Na
K
Cl
Ca
Al
Si
S
Zn
Concenration (μg·m⁻³)
22/3/2013
14/5/2013
12/6/2013
15/6/2013
8/11/2013
Date
(a)
(b)
(c)
(d)
(e)
(f)
(g)
(h)

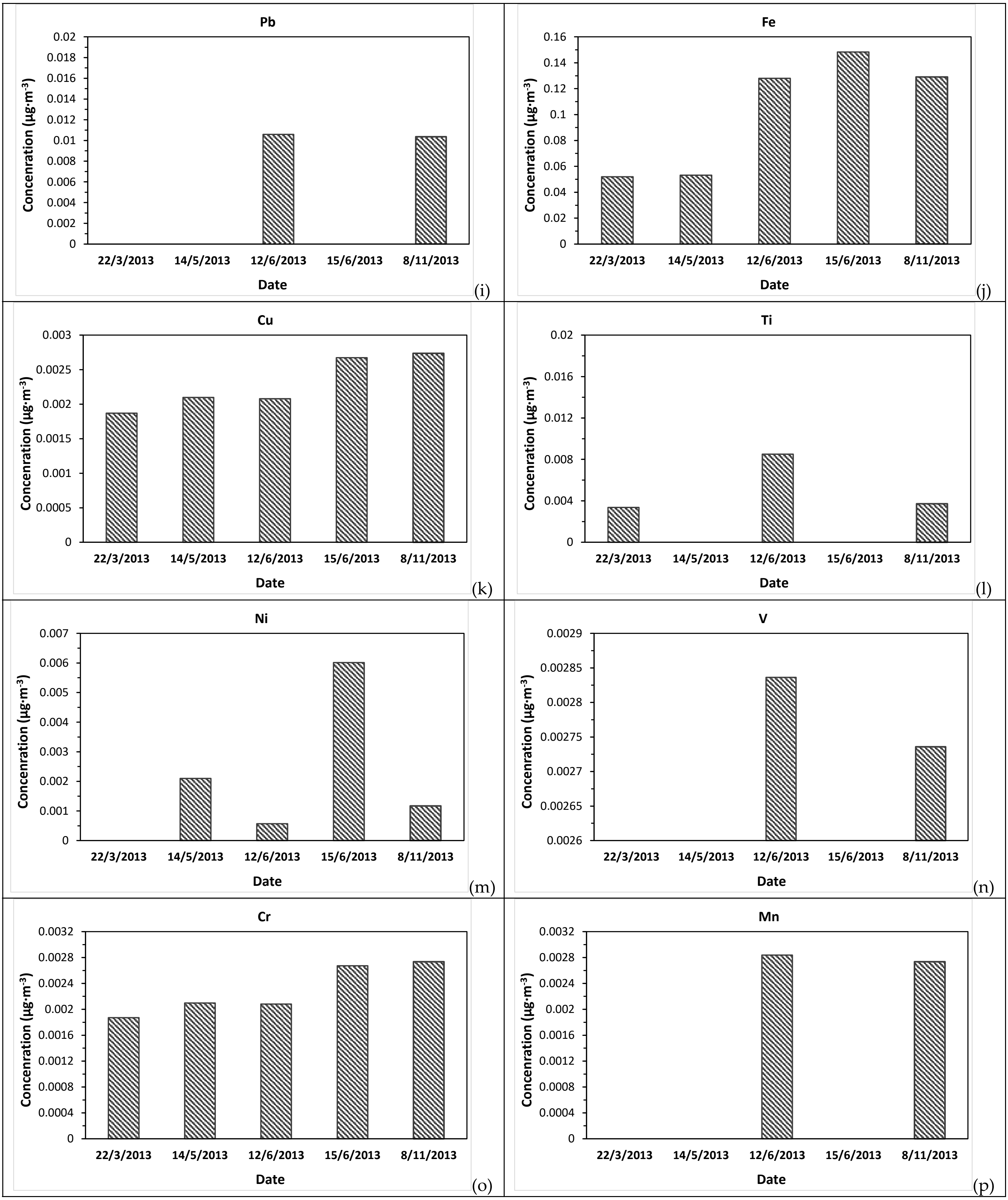
Pb
Fe
Cu
Ti
Ni
V
Cr
Mn
Concenration (μg·m⁻³)
22/3/2013
14/5/2013
12/6/2013
15/6/2013
8/11/2013
Date
0
0.002
0.004
0.006
0.008
0.01
0.012
0.014
0.016
0.018
0.02
0.04
0.06
0.08
0.1
0.12
0.14
0.16
0.0005
0.001
0.0015
0.0025
0.003
0.005
0.007
0.0026
0.00265
0.0027
0.00275
0.0028
0.00285
0.0029
0.0004
0.0008
0.0012
0.0016
0.0024
0.0028
0.0032
(i)
(j)
(k)
(l)
(m)
(n)
(o)
(p)

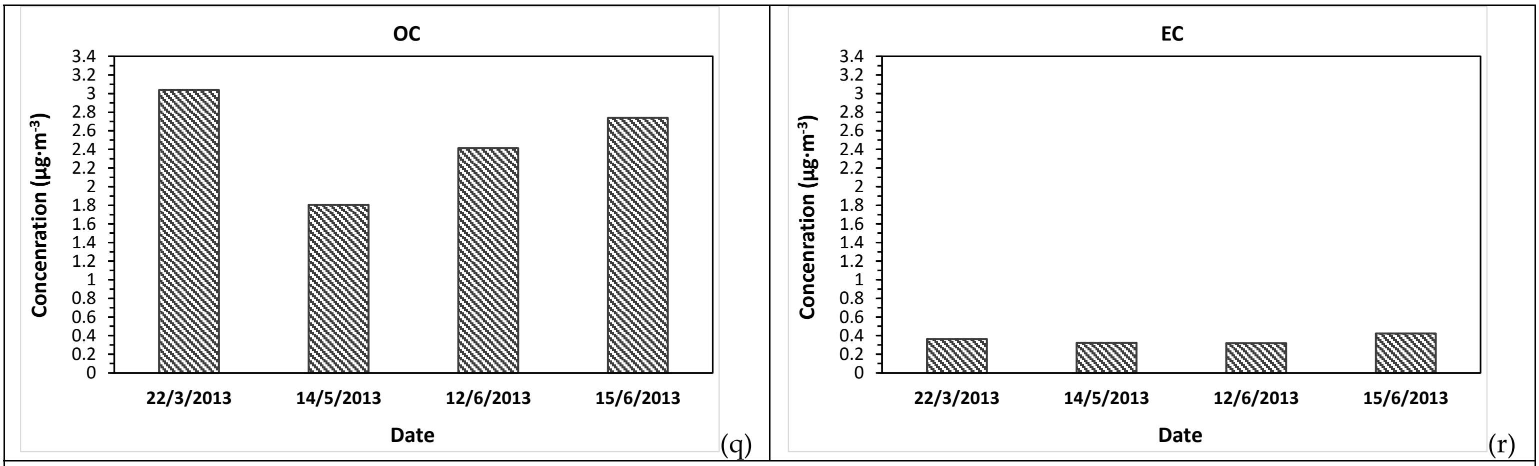

Figure S1. Concentrations of the detected chemical elements, organic carbon, and elemental carbon, which were adsorbed on the filters collected from the station at NCSRD throughout 2013.

Table S1. Statistical analysis of XRF measurements on the filters collected from the NCSRD station throughout the year 2013.

Chemical element	Mean	Median	Standard Deviation	Minimum	10th percentile	90th percentile	Maximum
Na	0.105	0.076	0.101	0.000	0.029	0.228	0.858
Al	0.097	0.041	0.208	0.003	0.010	0.154	1.563
Si	0.245	0.111	0.495	0.012	0.031	0.346	3.685
S	0.941	0.923	0.411	0.137	0.450	1.522	2.087
Cl	0.032	0.010	0.090	0.000	0.005	0.052	0.910
K	0.121	0.103	0.075	0.029	0.055	0.204	0.601
Ca	0.185	0.129	0.310	0.023	0.046	0.265	3.529
Ti	0.009	0.005	0.015	0.000	0.003	0.012	0.130
V	0.003	0.002	0.003	0.000	0.000	0.007	0.015
Cr	0.002	0.002	0.001	0.000	0.001	0.003	0.009
Mn	0.002	0.002	0.003	0.000	0.001	0.004	0.023
Fe	0.121	0.085	0.168	0.012	0.033	0.174	1.422
Ni	0.002	0.001	0.001	0.000	0.001	0.003	0.009
Cu	0.002	0.002	0.001	0.000	0.001	0.004	0.006
Zn	0.011	0.009	0.007	0.001	0.004	0.022	0.045
Pb	0.003	0.002	0.002	0.000	0.001	0.005	0.013
OC	2.88	2.64	1.24	0.36	1.42	4.38	7.59
EC	0.36	0.32	0.19	0.00	0.18	0.59	1.20

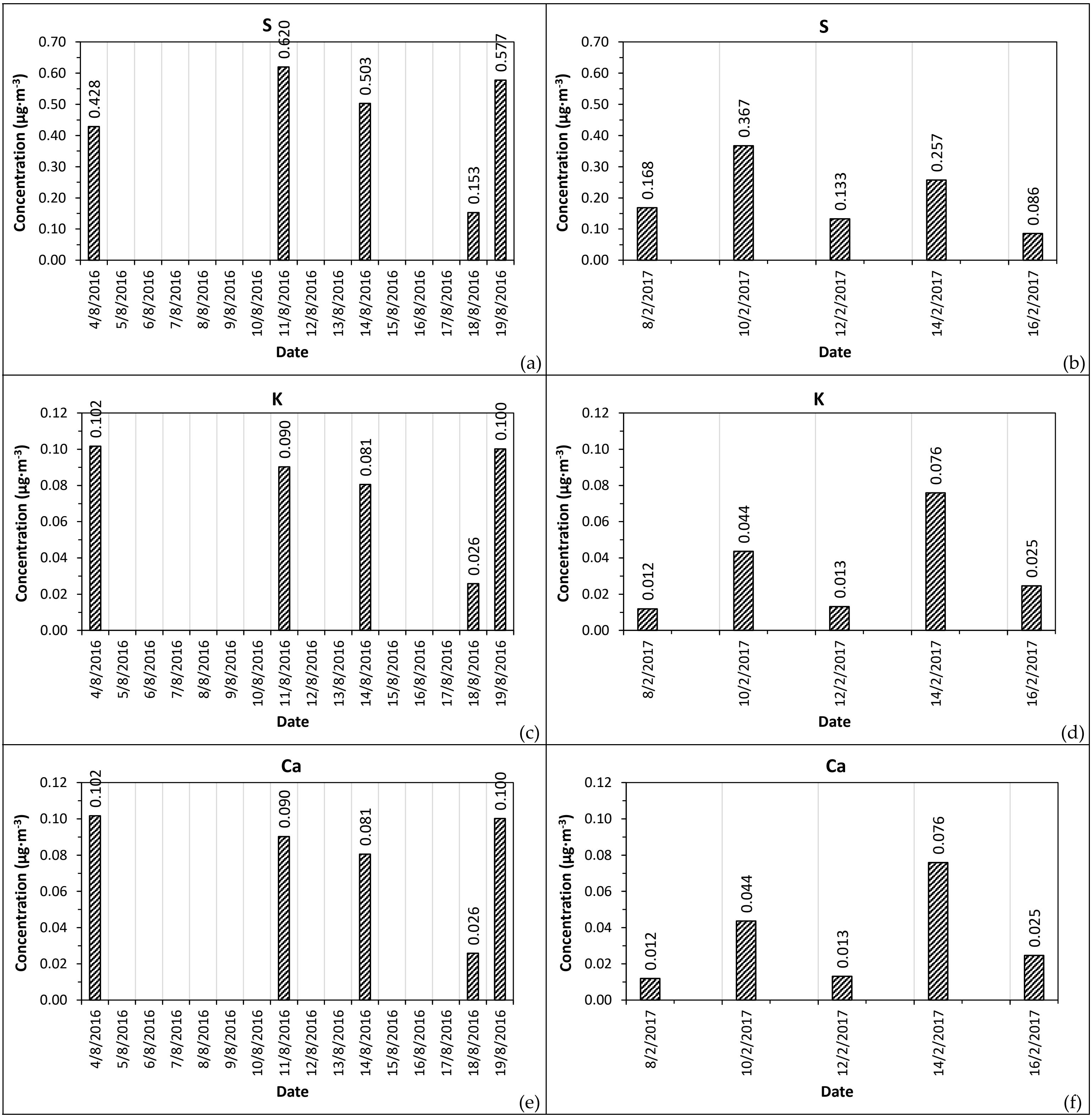
S
Concentration (µg·m⁻³)
0.00
0.10
0.20
0.30
0.40
0.50
0.60
0.70
0.428
0.620
0.503
0.153
0.577
4/8/2016
5/8/2016
6/8/2016
7/8/2016
8/8/2016
9/8/2016
10/8/2016
11/8/2016
12/8/2016
13/8/2016
14/8/2016
15/8/2016
16/8/2016
17/8/2016
18/8/2016
19/8/2016
Date
(a)
S
0.168
0.367
0.133
0.257
0.086
8/2/2017
10/2/2017
12/2/2017
14/2/2017
16/2/2017
Date
(b)
K
0.00
0.02
0.04
0.06
0.08
0.10
0.12
0.102
0.090
0.081
0.026
0.100
Date
(c)
K
0.012
0.044
0.013
0.076
0.025
Date
(d)
Ca
0.102
0.090
0.081
0.026
0.100
Date
(e)
Ca
0.012
0.044
0.013
0.076
0.025
Date
(f)

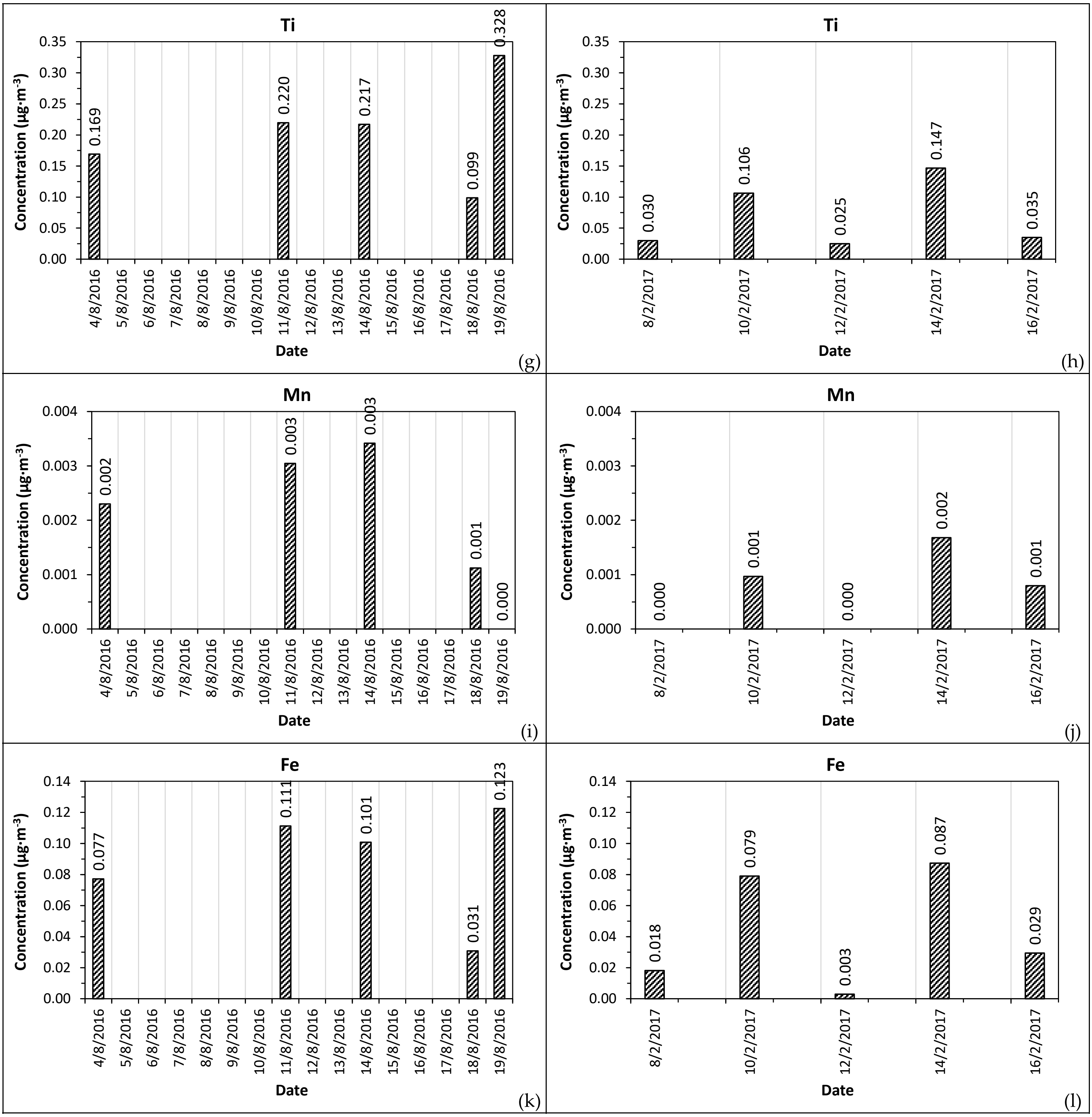
Ti
Concentration (µg·m-3)
0.169
0.220
0.217
0.099
0.328
4/8/2016 5/8/2016 6/8/2016 7/8/2016 8/8/2016 9/8/2016 10/8/2016 11/8/2016 12/8/2016 13/8/2016 14/8/2016 15/8/2016 16/8/2016 17/8/2016 18/8/2016 19/8/2016
Date
(g)
Ti
Concentration (µg·m-3)
0.030
0.106
0.025
0.147
0.035
8/2/2017 10/2/2017 12/2/2017 14/2/2017 16/2/2017
Date
(h)
Mn
Concentration (µg·m-3)
0.002
0.003
0.003
0.001
0.000
4/8/2016 5/8/2016 6/8/2016 7/8/2016 8/8/2016 9/8/2016 10/8/2016 11/8/2016 12/8/2016 13/8/2016 14/8/2016 15/8/2016 16/8/2016 17/8/2016 18/8/2016 19/8/2016
Date
(i)
Mn
Concentration (µg·m-3)
0.000
0.001
0.000
0.002
0.001
8/2/2017 10/2/2017 12/2/2017 14/2/2017 16/2/2017
Date
(j)
Fe
Concentration (µg·m-3)
0.077
0.111
0.101
0.031
0.123
4/8/2016 5/8/2016 6/8/2016 7/8/2016 8/8/2016 9/8/2016 10/8/2016 11/8/2016 12/8/2016 13/8/2016 14/8/2016 15/8/2016 16/8/2016 17/8/2016 18/8/2016 19/8/2016
Date
(k)
Fe
Concentration (µg·m-3)
0.018
0.079
0.003
0.087
0.029
8/2/2017 10/2/2017 12/2/2017 14/2/2017 16/2/2017
Date
(l)

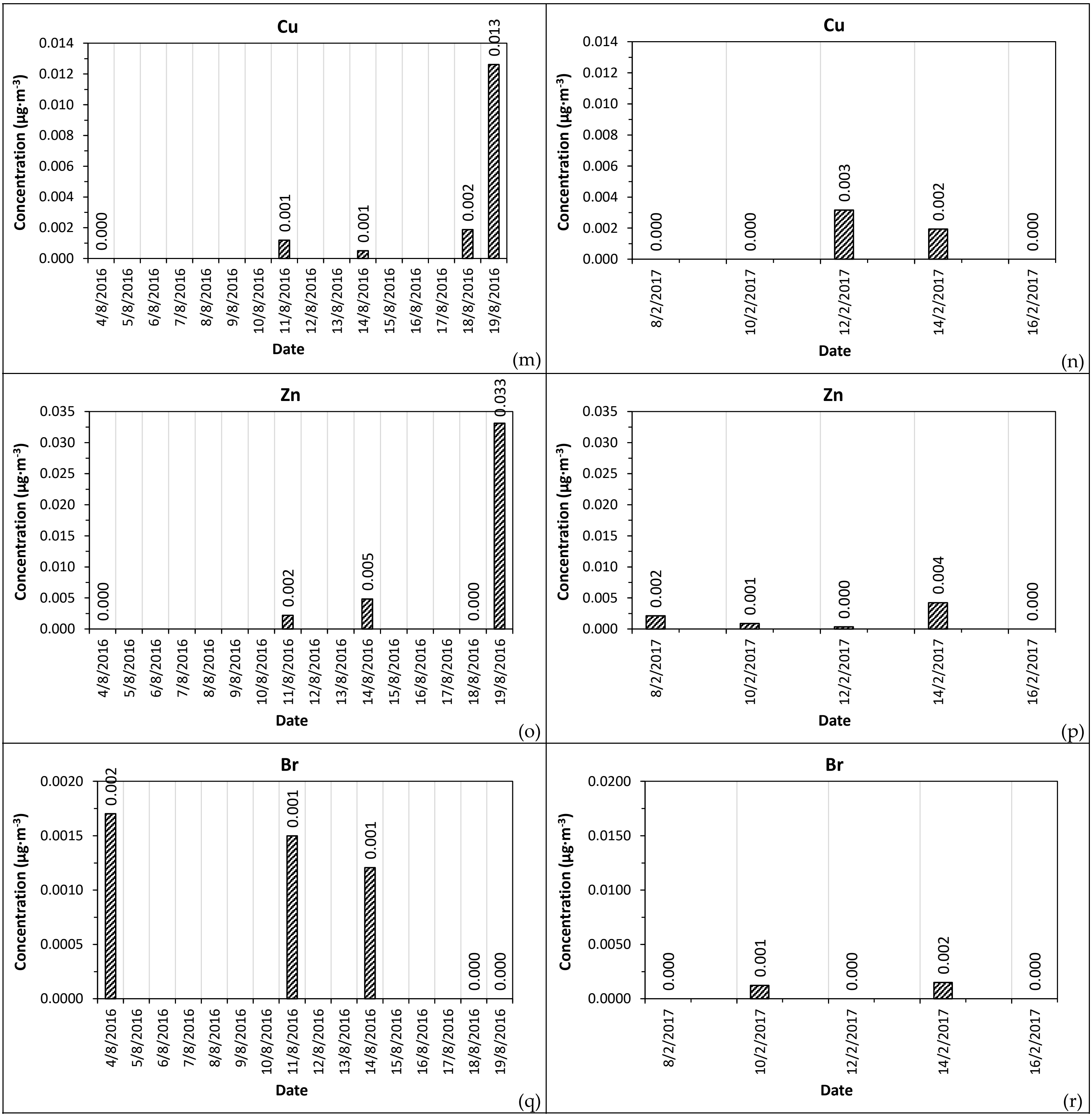

Cu
Concentration (µg·m⁻³)
0.014
0.012
0.010
0.008
0.006
0.004
0.002
0.000
0.000
0.001
0.001
0.002
0.013
4/8/2016
5/8/2016
6/8/2016
7/8/2016
8/8/2016
9/8/2016
10/8/2016
11/8/2016
12/8/2016
13/8/2016
14/8/2016
15/8/2016
16/8/2016
17/8/2016
18/8/2016
19/8/2016
Date
(m)
Cu
Concentration (µg·m⁻³)
0.000
0.000
0.003
0.002
0.000
8/2/2017
10/2/2017
12/2/2017
14/2/2017
16/2/2017
Date
(n)
Zn
Concentration (µg·m⁻³)
0.035
0.030
0.025
0.020
0.015
0.010
0.005
0.000
0.000
0.002
0.005
0.000
0.033
Date
(o)
Zn
0.002
0.001
0.000
0.004
0.000
Date
(p)
Br
Concentration (µg·m⁻³)
0.0020
0.0015
0.0010
0.0005
0.0000
0.002
0.001
0.001
0.000
0.000
Date
(q)
Br
Concentration (µg·m⁻³)
0.0200
0.0150
0.0100
0.0050
0.0000
0.000
0.001
0.000
0.002
0.000
Date
(r)

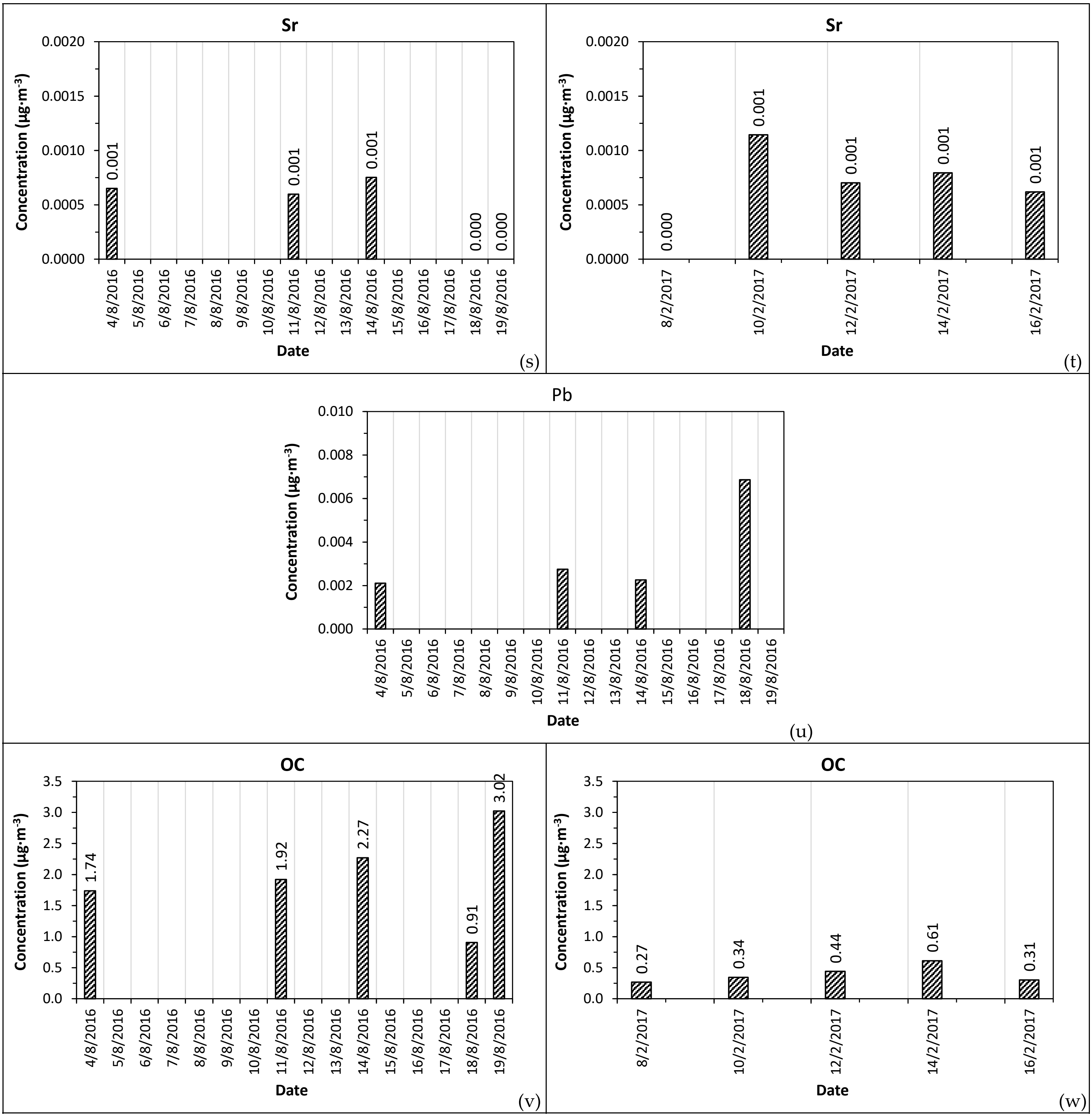
Sr
Concentration (µg·m⁻³)
Date
0.001
0.001
0.001
0.000
0.000
(s)
Sr
Concentration (µg·m⁻³)
Date
0.000
0.001
0.001
0.001
0.001
(t)
Pb
Concentration (µg·m⁻³)
Date
(u)
OC
Concentration (µg·m⁻³)
Date
1.74
1.92
2.27
0.91
3.02
(v)
OC
Concentration (µg·m⁻³)
Date
0.27
0.34
0.44
0.61
0.31
(w)

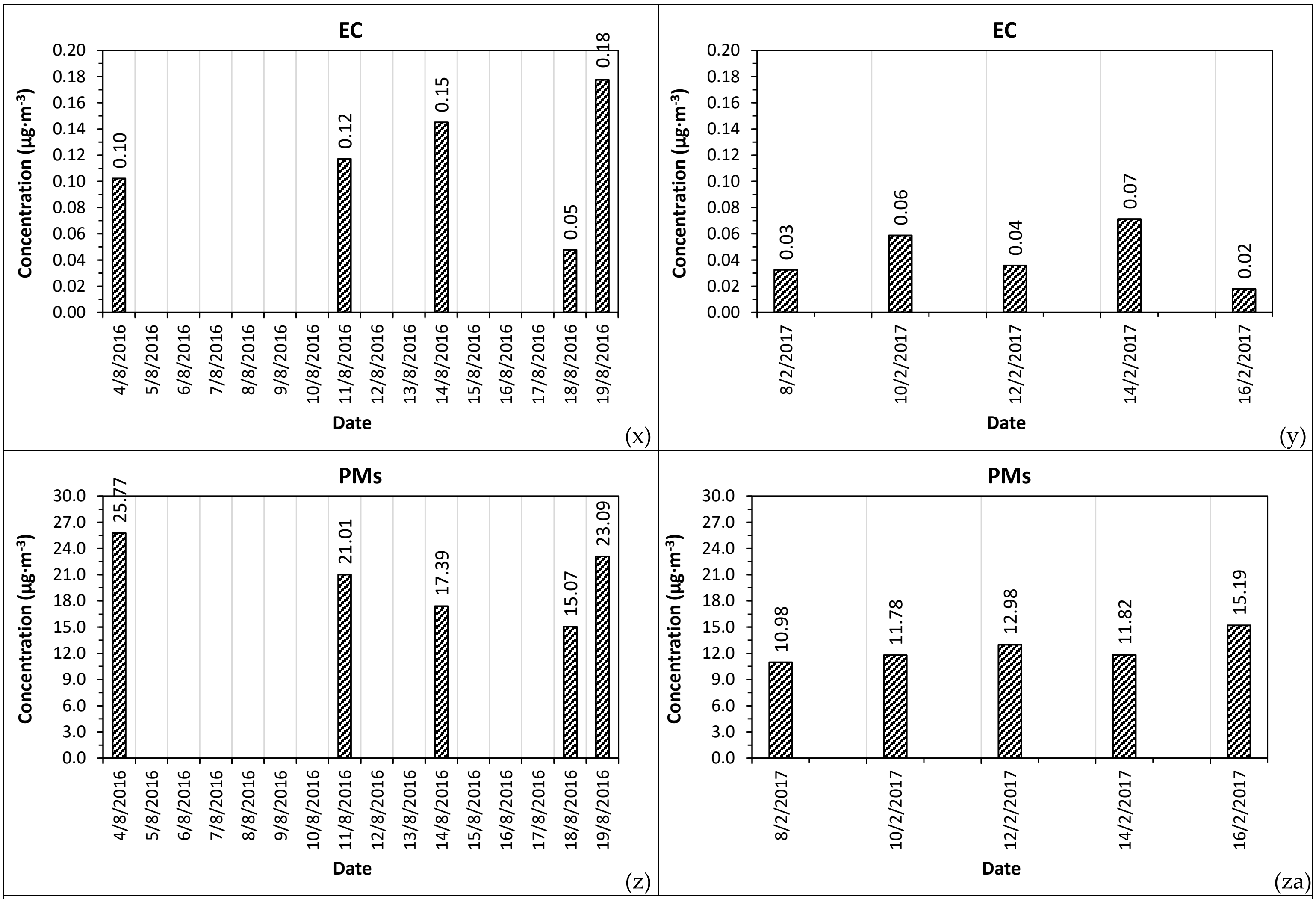

Figure S2. Concentrations of the detected chemical elements, organic carbon, elemental carbon, and $PM_{2.5}$, which were adsorbed on the filters collected from the station on mount Helmos during August 2016 and February 2017.

Table S2. Statistical analysis of XRF measurements on the filters collected from the mount Helmos station during 8/ 2016 and 2/2017.

Chemical element	Mean	Median	Standard Deviation	Minimum	10th percentile	90th percentile	Maximum
S	0.364	0.270	0.303	0.000	0.052	0.801	1.145
K	0.128	0.066	0.166	0.000	0.007	0.309	0.754
Ca	0.349	0.147	0.594	0.000	0.018	0.913	4.348
Ti	0.023	0.008	0.040	0.000	0.000	0.057	0.219
Mn	0.005	0.002	0.008	0.000	0.000	0.011	0.057
Fe	0.213	0.080	0.368	0.000	0.007	0.532	2.165
Cu	0.002	0.001	0.004	0.000	0.000	0.007	0.021
Zn	0.004	0.002	0.005	0.000	0.000	0.007	0.033
Br	0.001	0.001	0.002	0.000	0.000	0.003	0.026
Sr	0.003	0.001	0.005	0.000	0.000	0.006	0.031
Pb	0.003	0.000	0.006	0.000	0.000	0.007	0.029
OC	1.376	0.968	1.306	0.000	0.271	3.170	11.526
EC	0.103	0.078	0.101	0.000	0.018	0.195	1.136

Table S3. Variation of the supplied air volume, concentration and total mass of $PM_{2.5}$ and Fe, and fluorescence intensity per m^3 air, for the time intervals of 30 min and 45 min.

Demokritos station	Supplied air volume (m^3)	Concentration of $PM_{2.5}$ (μg/m^3 air)	Total mass of PMs (mg)	Fe concentration (μg/m^3 air)	Fe total mass in PMs (μg)	FU/m^3 air	
						30 min	45 min
22/3/2013	31.95	4.69	0.150	0.052	1.66	1323.36	303.88
14/5/2013	34.17	3.95	0.135	0.053	1.82	1921.36	1945.16
12/6/2013	63.2	5.7	0.360	0.129	8.15	4044.75	4724.49
15/6/2013	17.89	4.47	0.080	0.148	2.65	4427.00	7051.71
8/11/2013	61.15	6.21	0.380	0.128	7.83	7205.53	7430.47

Table S4. Variation of the supplied air volume, concentration and total mass of $PM_{2.5}$ and Cu, and fluorescence intensity per m^3 air, for the time intervals of 30 min and 45 min.

Demokritos station	Supplied air volume (m^3)	Concentration of $PM_{2.5}$ (μg/m^3 air)	Total mass of PMs (mg)	Cu concentration (μg/m^3 air)	Cu total mass in PMs (μg)	FU/m^3 air	
						30 min	45 min
22/3/2013	31.95	4.69	0.150			1323.36	303.88
14/5/2013	34.17	3.95	0.135	0.0105	0.36	1921.36	1945.16
12/6/2013	63.2	5.7	0.360	0.0136	0.86	4044.75	4724.49
15/6/2013	17.89	4.47	0.080	0.0220	0.39	4427.00	7051.71
8/11/2013	61.15	6.21	0.380	0.0149	0.91	7205.53	7430.47
